# A phylogenetic analysis of the grape genus (*Vitis* L.) reveals broad reticulation and concurrent diversification during neogene and quaternary climate change

**DOI:** 10.1186/1471-2148-13-141

**Published:** 2013-07-05

**Authors:** Yizhen Wan, Heidi R Schwaninger, Angela M Baldo, Joanne A Labate, Gan-Yuan Zhong, Charles J Simon

**Affiliations:** 1 College of Horticulture, Northwest A&F University, Yangling, Shaanxi 712100, People’s Republic of China; 2 US Department of Agriculture, Agriculture Research Service, Plant Genetic Resources Unit, New York State Agricultural Experiment Station, Cornell University, Geneva, NY 14456, USA; 3 US Department of Agriculture, Agriculture Research Service, Grape Genetic Research Unit, New York State Agricultural Experiment Station, Cornell University, Geneva, NY 14456, USA

**Keywords:** Biogeography, Divergence time estimate, Grapevine, Molecular phylogeny, Network, Northern hemisphere, Plant disjunction, Reticulation, SNP, *Vitis*

## Abstract

**Background:**

Grapes are one of the most economically important fruit crops. There are about 60 species in the genus *Vitis*. The phylogenetic relationships among these species are of keen interest for the conservation and use of this germplasm. We selected 309 accessions from 48 *Vitis* species,varieties, and outgroups, examined ~11 kb (~3.4 Mb total) of aligned nuclear DNA sequences from 27 unlinked genes in a phylogenetic context, and estimated divergence times based on fossil calibrations.

**Results:**

*Vitis* formed a strongly supported clade. There was substantial support for species and less for the higher-level groupings (series). As estimated from extant taxa, the crown age of *Vitis* was 28 Ma and the divergence of subgenera (*Vitis* and *Muscadinia)* occurred at ~18 Ma. Higher clades in subgenus *Vitis* diverged 16 – 5 Ma with overlapping confidence intervals, and ongoing divergence formed extant species at 12 – 1.3 Ma. Several species had species-specific SNPs. NeighborNet analysis showed extensive reticulation at the core of subgenus *Vitis* representing the deeper nodes, with extensive reticulation radiating outward. Fitch Parsimony identified North America as the origin of the most recent common ancestor of extant *Vitis* species.

**Conclusions:**

Phylogenetic patterns suggested origination of the genus in North America, fragmentation of an ancestral range during the Miocene, formation of extant species in the late Miocene-Pleistocene, and differentiation of species in the context of Pliocene-Quaternary tectonic and climatic change. Nuclear SNPs effectively resolved relationships at and below the species level in grapes and rectified several misclassifications of accessions in the repositories. Our results challenge current higher-level classifications, reveal the abundance of genetic diversity in the genus that is potentially available for crop improvement, and provide a valuable resource for species delineation, germplasm conservation and use.

## Background

Grapes (*Vitis* spp*.*) are one of the world’s most economically valuable fruit crops [1]. They are widely used for wine, table grapes, raisins, juice, and spirits; recent trends have also focused on antioxidants and healthful products derived from grapes. V*itis vinifera* L. subsp. *vinifera* (referred to as *V. vinifera* hereafter) is the most widely cultivated grape species but its productivity was historically limited due to its susceptibility to pests, diseases, and abiotic stress such as cold [2]. Genes from wild grape germplasm have been used to improve biotic and abiotic tolerance and resistance in cultivated grapes.

**Figure 1:**
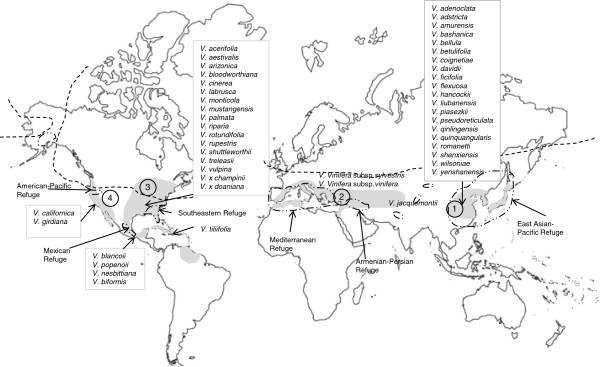
**Native geographic distribution of the genus *Vitis *(grey shading**^**1**^**) and geographic regions of origin of *Vitis *species used in this study.** Dashed lines indicate southern borders of the polar ice cap during the most recent ice age^2^. Dash-dot lines indicate ice age refugia of the forest flora^2^. Areas labeled 1 through 4 were used in ancestral area optimization (reversible parsimony, Additional file 14). Redrawn from ^1^Alleweldt et al. [7], ^2^Reinig [14].

Centers of grapevine diversity are found in the southeastern US [3-5] and East Asia [4,6]. Up to 30 species are native to a vast area in eastern Asia, China, Japan and Java, two species across middle Asia and Europe, and up to 28 species across the eastern and southwestern US and Mexico [2] (Figure 1). Appendix 1 expands discussion of the biogeographic background. The genus *Vitis* is divided into two subgenera: *Muscadinia* Planch. (2*n* = 40, one or two species) and *Vitis* Planch. (2*n* = 38, the remaining species). Additional divisions within *Vitis* are “series” which are subgeneric groupings that have been used historically in the systematics of *Vitis*. They rank below “sections” that are more commonly used in plant systematics for groups of species. Although all wild species are considered diploid, there is evidence of hexaploidization in their distant past [7-9] that is shared with all rosids [9]. The two subgenera are nearly reproductively isolated while the species within subgenus *Vitis* are interfertile. All species are dioecious except *V. vinifera* which has hermaphroditic flowers, and *V. rotundifolia* Michx. which segregates for this trait. Many species have overlapping distributions, thus natural hybridization would occur were it not for ecological and phenological barriers [3,10,11]. Not surprisingly, the classification of *Vitis* is confused in part due to the lack of agreement among systematic botanists as to what constitutes a true species and because of extreme morphological variation within the species [2,3,7]. This has led to many extraneous species names [7,12]. The systematics of *Vitis* is based primarily on morphology [13] and molecular methods have only recently been used to study this taxonomic problem.

Most previous molecular studies on the evolutionary history of *Vitis* were limited in taxonomic scope or marker choice [15-32]. Studies most similar in goals and pertinent to the present study were conducted by Aradhya et al. [33], Nie et al. [34], Liu et al. [35], and Zecca et al. [36]. Aradhya et al. [33] obtained a taxon sample similar to the present study and used SSR and AFLP markers to study genetic diversity within *Vitis*. These markers have limited value for phylogeny reconstruction [37] and dating divergences was not attempted. Nie et al. [34] and Liu et al. [35] provided well-reasoned paleontological dates to estimate divergences in Vitaceae. These calibration points were applied in the present study. Zecca et al.’s [36] chronogram is a tantalizing expansion of the *Vitis* component of Nie et al.’s [34] chronogram. Their inferences were limited by the small number of markers and the limited variability available in those markers, which did not fully resolve the tip clades. Further, limited intra-specific replication (sampling) limited the ability to make species-level inferences.

Adding more data can be useful for resolving difficult phylogenies that were based on a few genes [38]. The present study attempted to improve on three aspects of previous phylogeographical studies of *Vitis* by more extensive sampling of the nuclear genome, the species, and intraspecific variation. This study developed and used 27 nuclear gene markers and sequenced 309 accessions of 48 *Vitis* species, varieties, and four out-groups to: 1) reconstruct a phylogenetic hypothesis of the genus *Vitis*, 2) date important time points in the evolution of *Vitis*, 3) elucidate the biogeographic history of the genus, and 4) evaluate systematics of *Vitis* within the framework of phylogeny.

## Results

### Molecular characteristics of the nuclear sequences of *Vitis*

Most *Vitis* accessions had complete sequence or had minimal missing data (Additional file 1). Indel sequences in the 27 gene markers were unambiguous and easy to align. The starting alignment matrix for the 27 gene markers and all 309 accessions was 11,437 bp long. Gap coding for Maximum Parsimony added 304 characters. Amino acid coding sequence accounted for 5,690 nt, 3’ or 5’ untranslated regions for 4,074 nt, and introns for 1,036 nt (Additional file 2). Because Trees by New Technology (TNT) does not output the number of parsimony-informative characters, we report the number of unique site patterns from Bayesian Evolutionary Analysis by Sampling Trees (BEAST) and Randomized Accelerated Maximum Likelihood (RAxML). The 52-OTU matrix had 1,855 unique site patterns; the 273-OTU matrix had 2,510 unique site patterns. Their distributions among the gene markers are listed in Additional file 2. Under the uncorrelated log-normal relaxed molecular clock, estimated from three combined runs in BEAST and calibrated with three fossil dates, the mean rate of substitution (meanRate) in the data set was 8.249×10^-4^ per million years (Effective Sample Size (ESS) = 959) and a coefficient of variation (CV) of 0.896 (ESS = 1284). Dividing this rate by the size of the data set (11,437 nt), the average rate of substitution in this data set was 7.2 × 10^-8^ substitutions per site per million years.

### Extent of reticulation and network in *Vitis*

Ancestral polymorphism with subsequent lineage sorting is difficult to distinguish from reticulation based on a phylogenetic pattern [38-42]. In our study, six (22%) gene fragments showed significant tests for recombination based on the Phi Test [43]: fragments 1313 (P = 0.033), 1973 (P = 0.033), 2415 (P = 0.006), 5069 (P = 0.033), 7022 (P = 0.047), 7029 (P = 0.019). When these fragments were excluded, the concatenated matrix still showed recombination, as expected (P = 0.000), representing independent assortment of markers. Lanier and Knowles [44] found that, in species-tree estimation, the gain of accuracy from sampling additional loci and/or individuals always exceeded inaccuracies related to recombination. Thus, in the present work no genes were excluded from subsequent analyses based on evidence of recombination.

**Figure 2:**
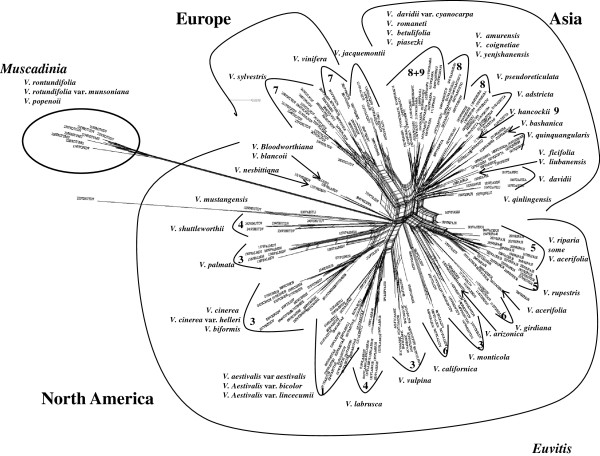
**The NeighborNet of 273 accessions based on 27 concatenated nuclear gene fragments.** Numbers indicate the series to which species have been recognized 1: *Aestivales* (Planchon); 2: *Cinerascentes* (Planchon); 3: *Cordifoliae* (Munson); 4: *Labruscae* (Planchon); 5: *Ripariae* (Munson); 6: *Occidentales* (Munson); 7: *Viniferae* (Planchon); 8: *Flexuosae* (Galet); 9: *Spinosae* (Galet). See also Additional file 15.

We used networks to better visualize the conflicts represented by the high levels of homoplasy. The consensus network [45] of the 26 individual gene trees indicated that few splits were common to multiple trees (Additional file 3). The NeighborNet [45] of the concatenated 273-OTU matrix (Figure 2) showed extensive conflict at the core of subg. *Vitis* representing the deeper nodes, and extensive conflict radiating outward.

### Estimation of divergence times in *Vitis*

**Figure 3:**
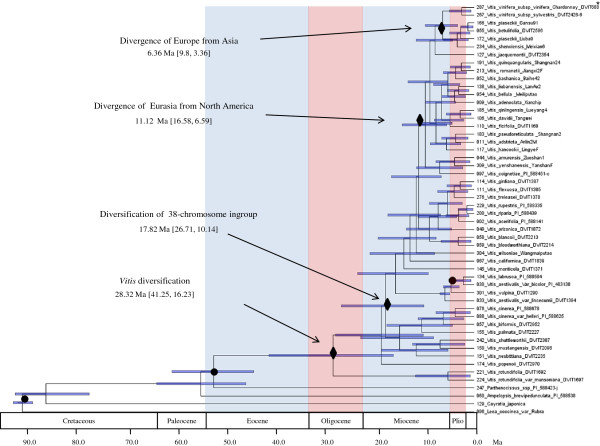
**Chronogram of Bayesian divergence time estimates of *Vitis *diversification based on 27 concatenated nuclear gene fragments inferred using the BEAST software.** Grey bars represent the 95% Highest Posterior Density (HPD) intervals of nodal age in million years. Calibration points are indicated with filled circles. Significant evolutionary events are indicated with black diamonds. Asterisk indicates inclusion of a clonally propagated cultivar that may affect the local divergence estimate. Additional files 4 and 5 show nodal ages and posterior probabilities for all nodes in this tree.

With the stem age of Vitaceae constrained at 90.7 ± 1.0 Ma, the stem age of *Vitis* at 58.5 ± 5 Ma, and the divergence of *V. labrusca* and its closely related North American relatives at 5.75 ± 0.5 Ma, the crown age of *Vitis* was estimated at 28.32 Ma (95% Highest Posterior Density (HPD) 41.25, 16.23), the crown age of subg. *Vitis* at 17.82 Ma (95% HPD 26.71,10.14), and the stem ages of most species fell between 11 and 1.3 Ma. The ten individual runs continued for 61.31 million to 89.21 million steps, and estimated the crown age of subg. *Vitis* between 17.285 Ma and 18.238 Ma. The Bayesian divergence times in Figure 3 were estimated from three combined, unpartitioned runs using BEAST. The maximum clade credibility tree with mean estimates of divergence time for all nodes (Additional file 4) and associated posterior probabilities (Additional file 5) were also obtained.

### Phylogenetic analyses in *Vitis*

#### Maximum likelihood

ML analyses of 26 single genes analyzed independently without missing data yielded 26 very poorly resolved trees. No significant conflict was observed under ML, thus the sequences were concatenated.

**Figure 4:**
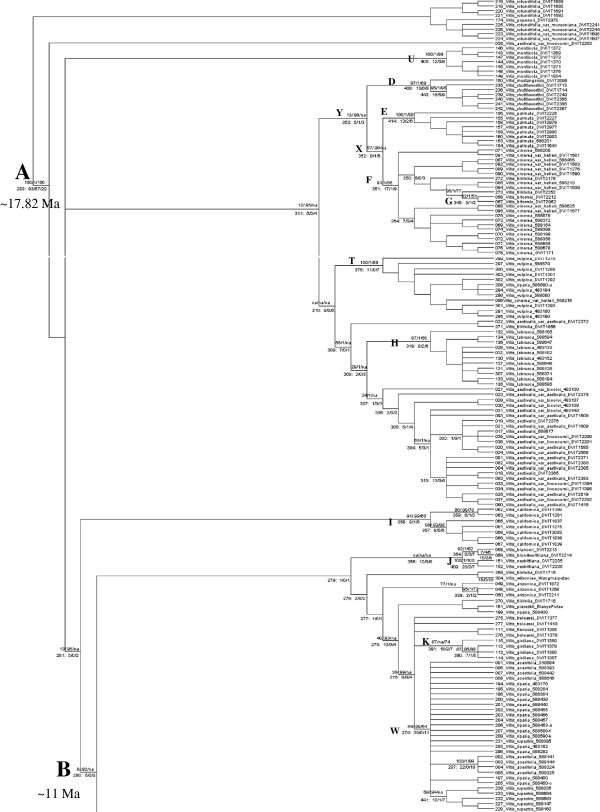
**Hypothesis of phylogenetic relationships among *Vitis *species.** North America. Strict consensus of 282 most parsimonious trees from four driven searches with support values for selected nodes. Above branches: (Maximum Likelihood BS/ Bayesian PP/ Maximum Parsimony BS). Values can range from 1-100 in ML, 0-1 in BA, 50-100 in MP. Below branches: (node number: branch length/No unique characters/No genes in support of node). The annotation “319: 8/2/6” means that node number 319 has 8 character changes of which 2 are unique to that node, and 6 genes contributed changes. “na” indicates absence of a value in the specific support category. Branch lengths, bootstrap supports, and posterior probabilities for all available branches are shown in Additional files 7, 8, 13, 16. Nodes labeled A-Y are discussed in the text. Figure 4 continues in Figure 5.

**Figure 5:**
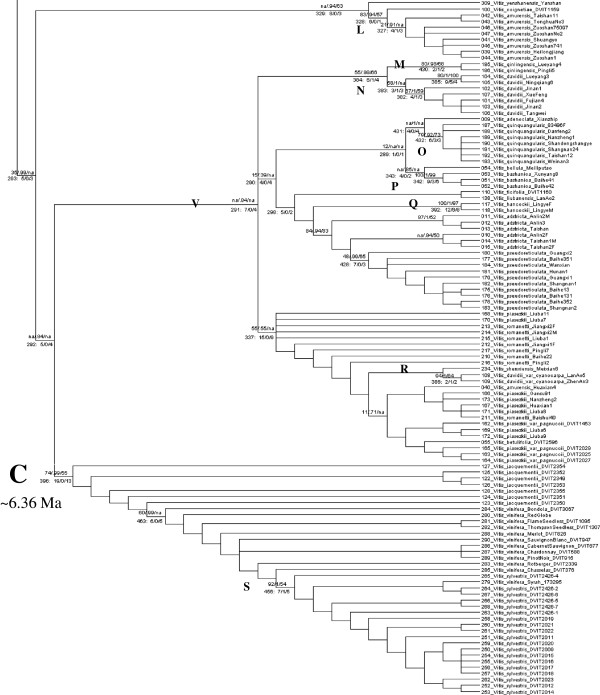
**Hypothesis of phylogenetic relationships among *Vitis *species.** Eurasia.Continuation of Figure 4.

The 20 identical (except for the starting seed) partitioned rapid ML runs using the 273-OTU matrix produced a range of maximum likelihood values of -42511to -42380. The final search on the same matrix following the bootstrap search yielded the highest likelihood value: -42358. This tree was used in comparisons with results from other methods using a cartoon, i.e., a simplified version with collapsed terminal clades (Additional file 6). Bootstrap values are reported for selected nodes (Figures 4 and 5) and all nodes (Additional file 7).

#### Bayesian

The two runs timed out at 48 mil and 50 mil generations with good effective sample size (ESS; > > 200), but they did not converge on the exact same phylogenetic hypothesis. Both trees were highly concordant for the species-level clades but differed on the specific placement of some clades (Additional files 8 and 9). Because the 48 mil run (Additional file 8) had a higher mean log likelihood of the cold chain (LnL) after burnin, the posterior probabilities from this run were used to summarize supports (Figures 4 and 5). Overall comparisons of relationships above the species level were facilitated using the cartoon of this tree (Additional file 10).

#### Maximum parsimony

**Figure 6:**
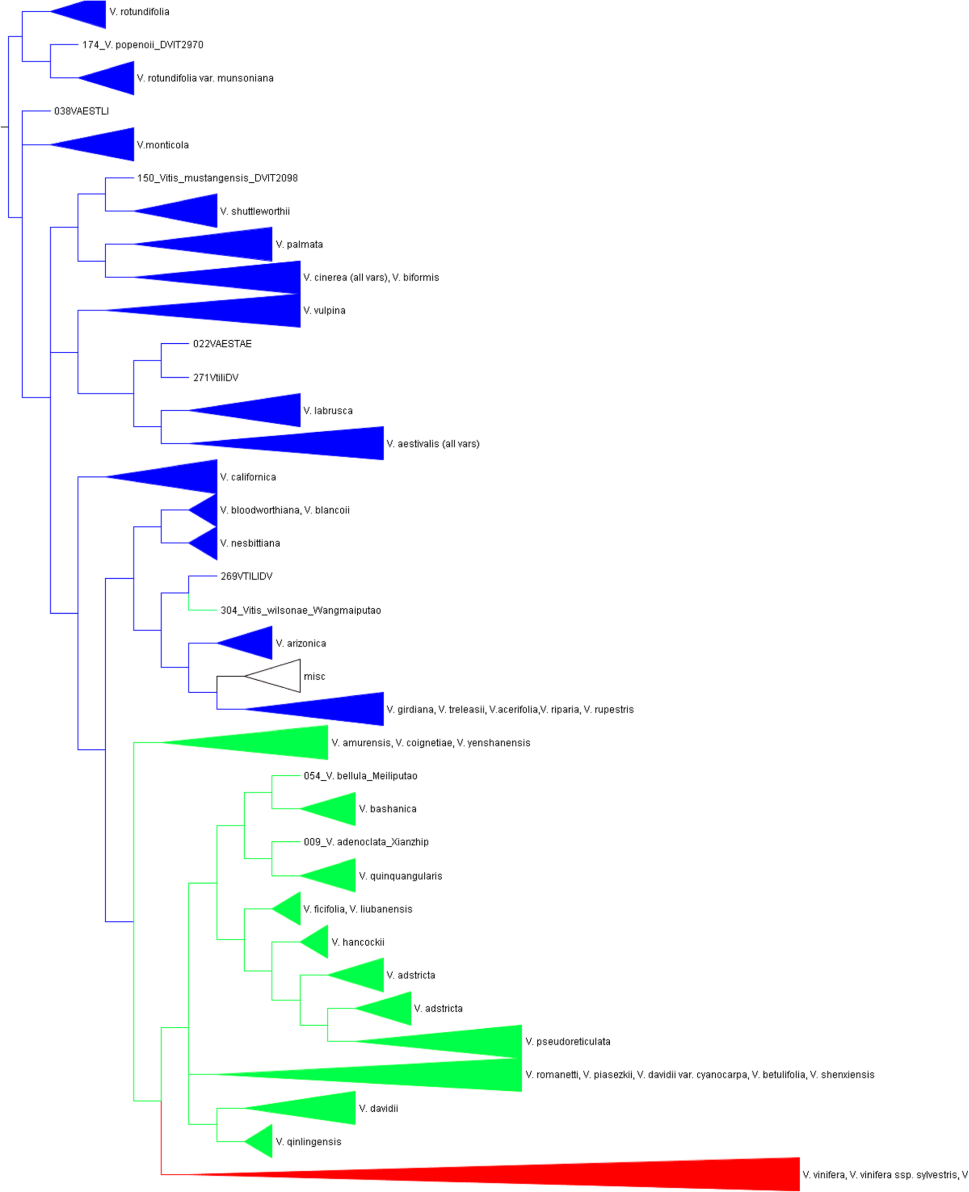
**Simplified version (cartoon) of the MP strict consensus tree.** Blue = North and Central American accessions, Green = Asian accessions, Red = European accessions. For comparison, Additional files 6 and 10 represent cartoons of the ML and BA trees, respectively.

TNT’s driven search function produced most parsimonious (MP) trees of the same length in all four searches. The individual MP trees had a score of 4398, consistency index (CI) of 0.360, and retention index (RI) of 0.790. The strict consensus of all 282 MP trees from all four searches had a score of 4580, CI = 0.346, and RI = 0.776. The strict consensus tree was simplified by collapsing terminal clades (Figure 6) for comparisons. The full tree was annotated with support values on selected nodes of interest (Figures 4 and 5, see figure legend). Node numbers are cross referenced with Additional file 11. All node numbers are shown in Additional file 12. The MP bootstrap tree with supports >50% was illustrated (Additional file 13).

#### Ancestral area analysis

Fitch parsimony identified Eastern/Southeastern North America (that also included Mexico and the Caribbean) as the origin of the most recent common ancestor of *Vitis* based on the strict consensus tree (Additional file 14).

### Systematics of *Vitis spp.*

It was evident that the patterns were similar among the cartoons of the strict MP consensus (Figure 6), the highest ML value cladogram (Additional file 6) and the Bayesian cladogram (Additional file 10). The clades of Eurasian species were nested in North America as a monophyletic clade. The series *Precoces* Munson (containing *V. riparia* Michaux, *V. acerifolia* Raf., *V. rupestris* Scheele) together with *V. arizonica* Engelm. (belonging to the series *Occidentales* Munson, Additional file 15), *V. blancoii* Munson*, V. bloodworthiana* Comeaux*, V. Xtreleasii* Munson ex L. H. Bailey and *V. girdiana* Munson formed the sister clade. *V. labrusca* and *V. aestivalis* Michx. were grouped together as were *V. cinerea* (Engelm.) Engelm. ex Millardet*, V. palmata* Vahl*, V. shuttleworthii* House and *V. mustangensis* Buckley. All three analyses did not group *V. monticola* Buckley or *V. californica* Benth. with other species.

The position of several clades differed among the searches. This was a notable characteristic of this data set. Among the different BA, ML and MP trees shown, the OTU composition of species clades in general was quite consistent and well supported, but a few clade or species positions were inconsistent. Correspondingly, clades above the species level were often poorly supported. For example, *V. yenshanensis* J. X. Chen was grouped with *V. amurensis* Rupr. and *V. coignetiae* Pulliat ex Planch. in MP and BA analysis and in the network but not in ML. Similarly, the *amurensis/coignetiae* clade was basal to Eurasia in MP and BA, but placed within the Asian clade in ML and in the network. *V. nesbittiana* Comeaux were grouped with *V. mustangensis* and *V. shuttleworthii* in BA and ML but grouped with *V. bloodworthiana* and *V. blancoii* in MP and in the network.

To better understand clade support, we further investigated the synapomorphies defining clades of interest. The characters supporting nodes of interest and their level of homoplasy and gene source were summarized based on MP (Additional file 11). Summary statistics for specific nodes investigated are shown below the branches in Figures 4 and 5. Many species showed good support with high bootstrap values and posterior probabilities, presence of node-specific characters and support from multiple genes (letters in parentheses refer to marked nodes in Figures 4 and 5): *V. shuttleworthii* (D), *V. palmata* (E), *V.cinerea* (all varieties and including *V. biformis* Rose*)* (F), *V. biformis* (G), *V. labrusca* (H), *V. californica* (I), *V. nesbittiana* (J), *V. girdiana* (K), *V. amurensis* (L)*, V. qinlingensis* (M), *V. davidii* (Rom. Caill.) Foëx(N), *V. quinquangularis* Rehd. (O), *V. bashanica* P.C. He (P)*, V. hancockii* Hance (Q), *V. davidii var. cyanocarpa* (Rom. Caill.) Foëx (R; and not grouping with *V. davidii*)*, V. vinifera* ssp*. sylvestris* (C. C. Gmel.) Hegi. (referred to as *V. sylvestris* hereafter) (S). The species *V. vulpina* L. (T) and *V. monticola* (U) each formed reliable species-level clades even in the absence of a species-specific SNP. Many higher level relationships were supported by a few characters and were of poor quality leading to the labile topology among major clades. Examples were the nodes defining the split between North America and Asia (B), Europe (with *V. jacquemontii* R. Parker) and China (C), and China without *V. amurensis* (V). These nodes had five to seven supporting characters that were frequently highly homoplasious (Figure 4; Additional file 11). The notable exception was the branch separating the *Muscadinia* from subg. *Vitis* (Figure 4 node A). It was supported by 93 characters of which 57 (61%) showed no homoplasy and represented 20 of the 27 (74%) gene markers. Other supported higher clades were the *V. cinerea*-*V. palmata* clade (X in Figure 4) and the (*V. mustangensis, V. shuttleworthii, V. palmata, V. cinerea* with *V. biformis*) clade (Y). Both nodes had a node-specific SNP, although bootstrap support and posterior probabilities were more consistent with other poorly supported higher-level clades.

The markers were informative in characterizing intra-specific variation in some species. The MP branch lengths (Additional file 16), MP and ML bootstrap supports and BA posterior probabilities (Additional files 7,8,13) supported intraspecific groupings well with non-zero branch lengths present in many species such as *V. shuttleworthii, V. monticola, V. californica, V. palmata, V. labrusca, V. cinerea, V. aestivalis, V. sylvestris, V. adstricta,* and *V. davidii*. Due to space constraints these supports were not summarized in (Figures 4 and 5).

## Discussion

### Problems of phylogenetic study in *Vitis*

Based on MP, ML, and BA phylogenetic reconstruction methods, the nuclear DNA dataset in this study had extensive variation to address genus-wide relationships in *Vitis*. The markers characterized intraspecific variation, defined most species, and strongly supported subg. *Vitis*. However, many of the relationships and deeper nodes (above species within subg. *Vitis*) were characterized by low bootstrap values and were often supported by few characters with high homoplasy. Low clade support and high homoplasy may be caused by insufficient data, parallel changes, reversals and convergences, as well as different histories of genes caused by lineage sorting and reticulation [40,46], and different clade sizes [47]. The ascertainment bias (the systematic distortion in measuring the true frequency of SNPs due to sampling) introduced in the marker development phase may have selected markers with insufficient variation outside *V. vinifera* or phylogenetic depth, and differences may be distorted due to domestication. However, we re-sequenced whole fragments, rather than genotyping *a priori* identified SNPs, thus included additional markers not restricted by our selection criteria of intermediate variability (see Methods). Further, the deepest node was very well supported both in number and quality of characters, illustrating that there was sufficient phylogenetic signal available to define subg. *Vitis*. Similarly, several species (e.g. *V. shuttleworthii*, *V. labrusca*, *V. palmata, V. hancockii, V. quinquangularis*) were well supported by multiple non-homoplasious characters. Thus, simple lack of data was not the definitive reason for poor support of deep nodes. Additional data, using the Vitis9KSNP array [22] or others in development, markers developed by Lijavetzki et al. [20] and Vezzulli et al. [30] or next generation sequencing (NGS) may resolve this phylogenetic problem. However, these data will certainly add more noise (homoplasious characters). In addition, ascertainment of homology in the data set created by NGS is very intractable because of complex paleopolyploidization and gene duplication in the grapevine genome [9]. It is possible that additional data from non-recombining chloroplast or mitochondrial DNA might add stable characters deeper within the tree. However, the literature [19,23,25,29,36,48] suggests that nucleotide substitution rates in these datasets may be too slow to add much intra-generic information. Species reticulation and incomplete lineage sorting would still present a challenge. Parallel changes, reversals, and convergence are likely minor contributing factors to the observed homoplasy due to the shallow phylogenetic depth of this study involving moderate levels of evolutionary time; these factors were further minimized by locus selection criteria.

### Ancestral polymorphism, reticulation and incomplete lineage sorting

Homoplasy due to incomplete sorting of ancestral alleles is more likely when the time between lineage splitting is short (short branch especially when deep in the tree [49]) and the effective population size is large [50]. The present estimates of divergence times showed that splitting events between the deeper clades occurred almost simultaneously within subg. *Vitis*. Myles et al. [22] found significant degrees of shared polymorphisms between North American wild grapevine species and European cultivated species, suggesting that grapevine species maintained large effective population sizes since their geographic isolation millions of years ago. Further, the linkage disequilibrium in *V. vinifera* is very low and haplotype blocks are very short [20-22,51], indicating significant historical recombination within the species [22]. There was significant recombination in several genes and in the concatenated dataset. Thus, the conflict in the NeighborNet (Figure 2) can be interpreted as evidence of shared ancestral polymorphisms mixed with reticulation and lineage sorting. The shared ancestral polymorphisms may be the cause of the central knot of conflict (an ancestral ocean of polymorphisms and reticulation) represented by the tight central mass of splits that represent incompatible and ambiguous signals in the data set [45], with the radiating splits representing progressive lineage sorting and reticulation within the lineages. Reticulating events include hybridization, recombination and horizontal gene transfer [45]. The first two were likely major factors in the evolution of *Vitis*, while horizonatal gene transfer was an unlikely mechanism. We conclude that extensive reticulation deep in the tree and incomplete lineage sorting are the likely reason for the lack of support at higher level nodes.

### Time frame of *Vitis* diversification

Our molecular dating is close to fossil estimates and distributional inferences that place the origination of *Vitis* into the Paleogene. The beginning of diversification among the extant taxa (crown age) in our tree was estimated at 28.32 Ma (95% HPD 41.25 Ma - 16.23 Ma). This was earlier than estimated by Nie et al. [34] who assessed it at about 8 Ma with a large 95% HPD, or Liu et al. [35] who assessed it at about 12 Ma (~22 Ma – 6 Ma), or Zecca et al. [36] with an estimated mean age of 18.60 Ma (28.79 Ma - 9.50 Ma) or 19.05 Ma (29.07 Ma - 10.2 Ma), depending on the details of their analyses. Estimates by all three [34-36] were predominantly based on (non-recombining) chloroplast sequence with 116, 1258, and 54 parsimony informative sites respectively, two studies [34,36] had one nuclear marker with 135 and 41 parsimony informative sites respectively, and each combined data set was <6000 nt, compared to the ~11,440 nt of the present study. Recombination in the present data possibly caused the estimate of the height of the tree to be greater, thus resulting in more ancient age estimates [44]. The use of the distant outgroup *Leea* may have caused problems in dating because of missing data and multiple substitutions. The inclusion of additional more closely related outgroups could improve the accuracy of the inferred dates [52]. The present estimate of 28.32 Ma appears a reasonable age considering that the estimate was associated with a large CI that reached back to 41 Ma and the oldest reliable *Vitis* seed date to the Paleocene (65.5 Ma - 55.8 Ma) [53,54]. The stem of *Vitis* did reach back that far (Figure 3). The diversification of subg. *Vitis* (Node A) was presently estimated at 17.82 Ma (26.71 Ma - 10.14 Ma), a node that was dated at 6.31 or 6.55 Ma (9.5 Ma -3.5 Ma, 9.34 Ma - 4.50 Ma) as node B in Zecca et al. [36], about 5.5 Ma (about 10 Ma - 2.5 Ma) by Nie et al. [34] and about 7 Ma (11 Ma - 5 Ma) by Liu et al. [35]. Higher level intra-North American divergences fell between 15.75 Ma -8.96 Ma. The divergence of Eurasia from North and Central America (the Asian- North American disjunction, node B in Figure 4) was 11.12 Ma (16.58 Ma –6.59 Ma). In Zecca et al. [36] this split coincided in timing with our Node A. Using sequence data of a single chloroplast gene (rbcL) and no fossil calibrations, Xiang et al. [55] estimated the divergence time of 11 East Asia-North American disjunct species, representing 11 diverse genera of flowering plants at an average of 4.98 Ma and ranging from 5.98 Ma to <0.28 Ma. Finally, we estimated 9.78 Ma to 5.28 Ma for higher level divergences among the extant taxa in Asia, and 6.36 Ma (9.8 Ma –3.36 Ma) for the separation of Europe and the Near East from Asia (Node C in Figure 5), a separation that was estimated by Zecca et al. [36] at 4.31 Ma or 4.47 Ma (6.60 Ma -2.31 Ma, 6.71 Ma – 2.61 Ma). We estimated species-level divergences between 11.68 Ma (*V. nesbittiana*) and 1.3 Ma (*V. piasezkii, V. betulifolia;* Additional file 4) and intraspecific divergences shown more fully in Figures 4 and 5 extended into more recent times. Overall, the timeframe estimated by the present study was more ancient than the estimates by other *Vitis*-specific molecular studies (Table 1).

**Table 1:** **Comparison of divergence estimates in *Vitis *among five studies that analyzed SNP data and used zero, two or three fossil calibration points**

**Node**^**1**^	**Xiang et al. 2000**^**2 **^[55]	**Nie et al. 2010**^**3 **^[34]	**Zecca et al. 2012**^**4,5 **^[36]	**Liu et al. 2013**^**6 **^[35]	**Present study**^**7**^
*Vitis* crown					
Estimate (Ma)	_^8^	~8	18.60, 19.05^5^	~12	28.32
95% HPD (Ma)	_	~16-4	28.79-9.50, 29.07-10.2	~22-6	41.25-16.23
subg. *Vitis* crown					
Estimate (Ma)	_	~5.5	6.31, 6.55	~7	17.82
95% HPD (Ma)	_	~10-2.5	9.50-3.50, 9.34-4.50	~11-5	26.71-10.14
Europe/Near East - Asia					
Estimate (Ma)	_	_	4.31, 4.47	_	6.36
95% HPD (Ma)	_	_	6.60-2.31, 6.71-2.61	_	9.80-3.36
Eurasia-NC America					
Estimate (Ma)	4.98 (5.98- < 0.28)	_	6.31, 6.55	_	11.12
95% HPD (Ma)	_	_	9.50-3.50, 9.34-4.50	_	16.58-6.59
Higher level intra North America					
Estimate (Ma)	_	_	_	_	15.75-8.96
95% HPD (Ma)	_	_	_	_	multiple
Higher level intra Asia					
Estimate (Ma)	_	_	_	_	9.78-5.28
95% HPD (Ma)	_	_	_	_	multiple
Species stem					
Estimate (Ma)	_	_	_	_	11.68-1.3
95% HPD (Ma)	_	_	_	_	multiple
Fossil calibrations (Ma), Priors used					
Vitaceae - *Leea*	_	85 ± 4.0^9^	85 ± 4.0	90.7 ± 1.0^10^	90.7 ± 1.0
*Vitis -Parthenocissus*	_	58.5 ± 5.0^11^	_	58.5 ± 5.0	58.5 ± 5.0
Old World - New world *Parthenocissus*	_	_	21.6 ± 6.4^12^	_	_
Within *Vitis*	_	_	4.5 ^13^	5.75 ± 0.5^14^	5.75 ± 0.5

^1^For nodes "Higher level intra North America", "Higher level intra Asia, "Species stem" the range contains several clades with different estimates, see Figure 3 and Additional file 4 for details, and the 95% HPD included multiple HDP associated with the several clades, see Figure 3. ^2^one chloroplast gene, 11 diverse Angiosperms; ^3^4226 nt (2794 cpDNA + 1432 nDNA); ^4^3583 nt (2701 cpDNA + 882 nDNA); ^5^estimates from analyses that either constrained the stem (first number) or the crown of subgenus *Vitis*; ^6^5471 nt cpDNA;^7^11,440 nt, 27 nuclear gene fragments; ^8^dash: information is not applicable; ^9^Wikström et al. [56] used a 3-gene data set, rate smoothing, and a single calibration point to estimate the stem age of Vitaceae at 78 - 92 Ma from which the prior distribution was derived; ^10^estimate from Magallón and Castillo [57] because Liu et al. [35] agree with arguments made by Nie et al. [58] that the previous Vitaceae stem age estimate [56] was inaccurate; ^11^stem age of the *Vitis-Ampelocissus* clade, located in the Paleocene to which the oldest reliable Vitaceae fossils date, including *Ampelopsis, Ampelocissus and Vitis*[35]; ^12^prior based on estimates by Nie et al. [35]; ^13^minimum age constraint based on fossil seeds described by Gong et al. [59]; ^14^based on fossil relatives of *V. labrusca* described by Gong et al. [59].

### Continental origin, dispersal and diversification of *Vitis*

The phylogenetic relationships and network of grapevines reflect the Northern hemisphere Cenozoic history. The extensive ancestral reticulations revealed by the network and analysis of individual genes suggested well connected ancestral populations and species throughout the distribution followed by increasing range-wide fragmentation, isolation, and differentiation. The ancestral area analysis and the recurring distributional trend of American paraphyly with Eurasia in this study suggested a progression from North America to Asia to Europe consistent with previous studies [17,29,33,36]. However, Péros et al. [23] concluded that their analysis may support an Asian origin of *Vitis*. Fossils of Vitaceae have been found frequently in Western North American Eocene deposits (55.8 to 33.9 Ma) and have not yet been found in southeastern localities [60]. Fossils of *Vitis* seed were found in deposits of the Rocky Mountains and Great Plains of North America [34,53] and in central Europe [34,54]. These findings assigned the oldest age of *Vitis* to the Paleocene (65.5-58.8 Ma). At that time the supercontinent Laurasia had only begun dividing into North America and Eurasia [61] and the climate was considerably warmer in the northern latitudes [62]. These factors facilitated dispersal of warm-temperate terrestrial organisms in the northern hemisphere. Most East Asia–North American disjuncts from diverse families have had longer histories in North America than in Asia: of nine woody East Asian–East North American disjunct genera [60] all appeared earlier in the fossil record of North America than in that of Asia [63]. Wen et al. [64] found many more lineages with North American origins and migration to Asia than *vice versa*[58]. Nie et al. [58] argue for a North American origin of *Ampelopsis* (Vitaceae). Molecular phylogenetic analyses of several disjunct genera suggested a progression from East Asia to Eastern and Western North America [65,66]. Thus, the balance of grape-specific information tends to support our findings of a North American origin for the most recent common ancestor of *Vitis*.

After fragmentation of a Paleo/Neogene range, our phylogenetic trees suggested isolation of some North American and Asian species during the Plio- and Pleistocene cooling cycles, post glacial range expansions, and ecological adaptation. Much of the current Eastern North American range of *V. riparia*, *V. labrusca*, *V. aestivalis,* and *V. cinerea* was unsuitable for *Vitis* during the Wisconsinan glaciations due to coverage by the polar ice sheet and harsh conditions along its southern edge (Figure 1). These species must have expanded to their large present ranges after the glacial period. Large range expansions with post glacial warming were also promoted by the physiographic homogeneity of Eastern North America [63]. Fragmentation and local adaptations were evident in physiographically heterogeneous western North America and temperate eastern Asia. The North American species *V. shuttleworthii*, *V. nesbittiana*, *V. girdiana*, *V. palmata*, *V. bloodworthiana* and *V. blancoii* have smaller ranges and multiple species-specific SNP character changes. Similarly, physiographically diverse eastern Asia [63] had three species with multiple species-specific SNPs: *V. bashanica, V. hancockii, V. quinquangularis*. Local adaptations in heterogeneous environments likely lead to smaller population sizes and thus more rapid loss or fixation of novel characters [67].

The underlying evolutionary scenario for *Vitis* is consistent with origin in the Eocene, a time of maximum development of temperate Paleo/Neogene forests. This was followed by diversification in the mid-Oligocene, the rise of subg. *Vitis* in the early Miocene, the North American and Asian disjunction in the late Miocene, range restriction and fragmentation and speciation during the Pliocene and Pleistocene cooling cycles. These caused the primary divisions within *Vitis* as well as species-level and some intra-specific divisions [68]. The North Atlantic land bridge was present in the early Paleogene [69,70] and may have no longer existed when *Vitis* arose, leaving Beringia as the major route for potential gene flow. The area of the Bering and Chukchi seas lay above sea level for most of the last 50 to 60 M years [71] and was suitable for exchanges of temperate plants [69] until the establishment of the Bering Seaway 3.5-5 Ma [72], permitting genetic exchange at least until late Miocene to which the disjunction was timed. The Pleiocene/Pleistocene cooling cycles are well known to have caused range restrictions, survival in refugia, and diversifications in many groups of organisms [73], both on land and in the sea. This study shows clearly that *Vitis* was also a part of this great biogeographic phenomenon.

### Phylogenetically-based *Vitis* systematics

The systematics of *Vitis* is a challenging area of taxonomy. Our findings confirmed the tenuous nature of many grapevine species and especially higher groupings such as series. The apparent species-specific SNPs are good candidates to apply in species delineation investigations of grapes.

The present study found very low support for all series that included more than one species except for the Munson/Moore series Precoces/Ripariae (Figure 4, node W). Other well supported higher-level groupings were subg. *Vitis* (Figure 4, node A) and genus *Vitis*, supporting the division of the genus *Vitis* into two sections [4,74,75] or subgenera [5,76]. Additional file 15 lists a synopsis of the major *Vitis* classifications. Only Galet [4] assigned Asian species to series. The most comprehensive treatment of Chinese *Vitis*[6] did not apply a series-level classification. Most Chinese species could be assigned to one series if series were to be used (Figure 5). This may not include *V. amurensis*, *V. coignetiae* and *V. yenshanensis* as these species in some analyses grouped firmly within Asia (as opposed to Figure 5 where they are basal to Eurasia). It appears as if *V. jacquemontii* should be assigned to the series *Viniferae*. However, our accessions had perfect flowers, suggesting past hybridization with *V. vinifera*. The phylogenetic position intermediate between the Asian and Eurasian species and the well-defined split revealed in the network (Figure 2) supported this conclusion.

The derived position of *V. sylvestris* was unexpected. *V. sylvestris* is the suggested progenitor of *V. vinifera*[77] while the phylogenetic position suggests that *V*. *sylvestris* was derived from *V. vinifera* (Figure 5). This may be an artifact of the tenuous nature of most higher-level relationships revealed in this study. It could also be a result of the nature of selection and clonal propagation that all *V. vinifera* cultivars included in the present study have been subjected to, some of them potentially for thousands of years [77,78]. Evolution is arrested by clonal propagation, leaving the naturally evolving wild species to appear more derived. Myles et al. [21] concluded that current commercial *V. vinifera* varieties are only one or two generations removed from the wild *V. sylvestris*.

Mullins et al. [10] hypothesized Asian/North American sister species pairs for *V. coignetiae/V. labrusca* and for *V. jaquemontii/V. tiliifolia* (*V. lanata* and *V. caribaea* in [10]). Our results did not support sister pair relationships for *V. coignetiae /V. labrusca* as these species placed solidly into well separated Asian and North American clades, respectively. Our results are inconclusive with respect to the *V. jacquemontii/V. tiliifolia* pair due to the possible hybrid nature of *V. tiliifolia* accessions in general and the dispersed positions of *V*. *tiliifolia* samples.

*V. girdiana* has been considered to be a variety of *V. arizonica*[75], a variety of *V. californica*[79] and its own separate species [75]. Our results preliminarily identified *V. girdiana* as a well supported independent species (using the general lineage concept [80] and diagnosability [81]) with five species-specific SNPs. More samples need to be investigated to assess the discriminatory power of these SNPs. Wada [31] also identified a monophyletic *V. girdiana* cluster, although it had poor bootstrap support.

Samples 080-084 came to us as *V. cinerea* (Engelm.) Engelm. ex Millardet var. *floridana* (Munson) but placed solidly into the *V. aestivalis* clade. This highlighted confusion in the past related to the synonym *Vitis simpsonii* that has been claimed for two different species as described in Comeaux [82], one belonging to Aestivales and the other to Cinerescentes. The synonym *V. rufotomentosa* has the same problem. Our study showed conclusively that these accessions belong to *V. aestivalis*. Several additional accessions were identified as misnamed and others were recognized as hybrids (Additional file 1). Finally, two accessions, 111*V*. *flexuosa* DVIT1385 and 304*V. wilsoniae* Wangmaiputao, were of Asian origin yet grouped with North American accessions and remain anomalies that could not be resolved.

## Conclusions

This is the first study to apply sequences of a large number of nuclear loci combined with extensive species and intraspecific sampling to the phylogeny and biogeographic history of *Vitis* and the problem of *Vitis* systematics. The genome-wide sampling of SNPs provided insight into the evolutionary history of the grape genus and supported previous notions of Paleogene origins, range fragmentation, and recent nature of the species, joining *Vitis* with the large group of organisms whose extant species differentiated in response to Pliocene and Quarternary climate change [73]. We found that the most recent common ancestor of *Vitis* was North American. The major clades formed throughout the native distribution at 23-8 Ma (broad range due to large HPDs), suggesting that vicariance (the fragmentation of a large Paleo/Neogene Northern hemisphere distribution) in conjunction with local adaptation, was a dominant force in structuring genetic diversity of extant *Vitis spp*. We demonstrated that genome-wide nuclear SNPs were a productive approach to address questions at and below the species level in grapes. Many species were well supported, and the markers with low homoplasy defining those lineages will likely be useful in species delineation and assessing the reliability of different morphological taxonomic characters. Most higher-level relationships within the genus suffered from weak support. The genus itself was extremely well supported. This suggested that the phylogenetic signal was too weak to overcome the level of noise created by evolutionary forces acting within the *Vitis* gene pool. Two of the most important forces, probably acting concurrently or alternating, are incomplete lineage sorting of ancestral polymorphism and reticulation. Broad reticulation across many species probably prevented the ancestral gene pool from diverging during the Neogene forest stage, maintained reproductive compatibility, and is still acting today as evidenced by the prevalence of hybrids found in the wild and in repository collections. However, climatic oscillations during the Pliocene and Quaternary, coupled with physiographic heterogeneity, provided enough recent barriers to gene flow to facilitate evolutionary divergence. In light of the recency of divergence and diffuse genetic boundaries, higher-level taxonomic groupings, such as series, may be misleading.

## Methods

### Plant materials

A total of 309 accessions of 48 species or varieties (~80% of the approximately 60 known species of the genus) and outgroups were sampled in this study: 21 species from Asia, both European species, and 25 species and varieties from North America (Figure 1; Additional file 1). These samples were obtained from: 1) the Grape Germplasm Collection at the Northwest A&F University (NAFU), Yangling, Shaanxi Province, China, (DNA), 2) USDA-ARS, Plant Genetic Resources Unit (PGRU), Geneva, NY, USA, and 3) USDA-ARS, National Clonal Germplasm Repository (NCGR), Davis, CA, USA. Four closely related genera based on chloroplast and nuclear markers [19,28,32,34] were chosen as outgroups in the dating of divergences using BEAST: *Parthenocissus* spp. Planch, *Ampelopsis glandulosa* (Wall.) Momiy. var. *brevipedunculata* (Maxim.) Momiy, *Leea coccinea* Planch. ‘Rubra’, *Cayratia japonica* Thunb. Two of the outgroup genera (five species of *Cissus* and *Cayratia japonica*) were obtained from a research collection (Dr. P. Cousins, USDA/ARS, presently E & J Gallo Winery). The outgroup *Leea coccinea* “Rubra” was grown from seeds obtained from Carter Seeds (Vista, California).

No cultivars of *Vitis spp.* were included except for *V. vinifera* ssp. *vinifera* for which no wild accessions are known [77]. To mitigate long-branch attraction, the 40 chromosome *Vitis rotundifolia* Michx. subg. *Muscadinia* was used as the outgroup in analyzing subg. *Vitis*. This was justified by all preliminary analyses on the complete data set that identified *V. rotundifolia* as the sister species to subgenus *Vitis* and it is consistent with other studies e.g. [23,29,33-36]. One to 27 accessions or genotypes were sampled per species. All available varieties (not cultivars) of a species were sampled. Similarly, widely distributed species were more extensively sampled to include potential geographic differentiation. A few sibling groups were also included to test the ability of the markers to place or distinguish those accessions.

Based on preliminary analyses, accessions that placed in unexpected positions or had very weak support on preliminary phylogenetic trees were submitted to a taxonomic expert (Dr. P. Cousins) for an independent assessment of species identity, but without indicating the nature of the conflict. Additional file 1 lists the results of all assessments. The labels in the figures and tables indicate the corrected names unless otherwise indicated. Exact geographic coordinates of origin were not available for most accessions. Accessions and pertinent details are listed in Additional file 1. The accessions located in the US repositories can be requested through the Genetic Resources Information Network (GRIN) [83] and plant materials (leaves, cuttings) can be requested from the clonally maintained vines at these sites.

### DNA isolation and re-sequencing

DNA was isolated from fresh or frozen young leaves and apical meristems using a modified CTAB (cetyltrimethylammonium bromide; Sigma H6292) protocol [84,85] with 2-5% PVP (Polyvinylpyrrolidone, mol. wt. 40,000; Sigma PVP40) in the extraction buffer to remove secondary compounds, two chloroform purifications to remove proteins and a NaCl and ethanol precipitation to remove polysaccharides.

Primer screening was performed in 25 μL PCR volumes. 50 μL PCR volumes were cleaned for sequencing, concentrated and used in 12 μL cycle sequencing reactions. Additional file 17 provides the detailed conditions.

The exploratory sequencing was performed in-house at PGRU on ABI-3100xl Genetic Analyzer. The high-throughput sequencing (30 gene fragments for 309 accessions) was performed by Genaissance Pharmaceuticals, Inc (New Haven CT, USA). Both strands were separately sequenced using the PCR forward or reverse primer.

### SNP discovery and selection

Expressed Sequence Tags (ESTs) [86] of *Vitis vinifera* and grape mRNAs in NCBI in 2004 were sub-clustered and surveyed to predict SNPs using an in-house pipeline as described by Labate and Baldo [87]. The 62 variably-sized EST libraries and additional grape mRNAs included 108,429 *V. vinifera* sequences which formed 3,792 clusters. Because EST data are often based on one sequencing pass and are not filtered for error, a predicted SNP may not be verifiable. Because this study was intended to survey broadly across the entire genus, gene markers that were predicted to be monomorphic among *V. vinifera* were discarded. Markers with extreme levels of polymorphism were also excluded to minimize possible selection of duplicated loci.

Pairs of PCR primers were designed using the program ‘Primer 3’ [88] for 281 gene fragments of 400-600 base pairs (bp) containing moderate polymorphism. The amplifications were tested using three DNA samples, one each from Asia (*V. romanetii* Rom. Caill. ‘Jiangxi2’), Europe (*V. vinifera* ‘Rotberger’, DVIT2339) and North America (*V. rotundifolia*, DVIT1689). Robust, single bands were obtained for 201 of 281 primer pairs (71.7%). Then 96 primer pairs with robust single bands were chosen for re-sequencing to test sequence quality using eight species (*V. cinerea* (PI588575), *V. labrusca* L. (PI588194), *V. amurensis* (Zuoshan1), *V. quinquangularis* (Weinan3), *V. romanetii* (Pingli7), *V. davidii* (Xuefeng), *V. hancockii* (Lingye_F) and *V. yenshanensis* (Yanshan_F). Thirty of the most consistently amplifiable gene fragments, both within *Vitis* and outgroups, with suitable polymorphisms and only minor sequence length variation in the eight tested, were re-sequenced in a total of 309 accessions (Additional file 1). Predicted genes were identified in comparison with the NCBI non-redundant protein sequence database. When the *V. vinifera* genome sequence became available [9], the primer sequences and gene fragments were BLASTed [89] against this genome to determine their chromosome locations and confirm their homology and identity. When the final dataset was assembled, three gene markers were excluded because of unalignable indels (one marker), and suspected duplicate loci (two markers). The sequences of the 27 final primer pairs and supporting information are listed in Additional file 2.

### Sequence alignment, data sets, coding of gaps

ProSeq [90] was used for editing sequence based on trace files, and Mutation Surveyor (Soft Genetics) was used for base calling. Heterozygotes were manually edited to use the IUPAC-IUB symbols for nucleotide nomenclature [91]. The results from ProSeq and Mutation Surveyor were compared for accuracy, and nearly 100% agreement was found. Discrepancies were resolved by examining trace files manually. Sequences were aligned manually and also aligned using Clustal W [92] with default parameters.

Extensive preliminary phylogenetic and PCA [93] (Additional file 18) analyses using all (Additional files 19, 20, with partitions listed in Additional file 21) and subsets of OTUs revealed known and new hybrids which were excluded in the final analysis because phylogenetic trees can be strongly influenced by hybrid taxa [94]. This does not guarantee that the final analyses were devoid of hybrids as they are not always identifiable based on morphology. The final phylogenetic data set contained 273 OTU composed of subgenera *Vitis* and *Muscadinia* (Additional files 1, 19, 20). This dataset was modified for dating divergences using BEAST: 1) Four outgroup taxa were added to match calibration points in Nei et al. [34]: 060_*Ampelopsis brevipedunculata*, 096_*Leea coccinea* ‘Rubra’, 129_*Cayratia japonica*, 247_*Parthenocissus* spp.. *Leea* and *Cayratia* had substantial amounts of missing data (Additional file 1). 2) With the presence of multiple individuals per species dating is a more complex issue and would preferably apply coalescence methods. Preliminary analyses indicated that the present data set was not sufficiently informative to allow a well-supported coalescent analysis. Thus, the number of ingroup OTUs was reduced to one accession per species and variety for efficient calculations [95] and to satisfy the Yule model of speciation. These modifications resulted in the 52-OTU dataset (Additional file 22). Additional file 1 summarizes the members of each analysis. Preliminary ML analyses were conducted on all single gene fragments, and partitioned and unpartitioned concatenated sets for a total evidence dataset [96].

Gaps were treated as characters using ‘simple indel coding' (SIC) [97] and implemented in SeqState 1.4.1 [98]. Simple gap coding was chosen because it is a preferred coding method for empirical studies [99,100]. Inclusion of gaps in phylogenetic analyses is limited by the optimality criterion used for phylogenetic inference. Gap information was used for parsimony analysis only. ML and BA treated gaps as missing data. Combinability of DNA partitions was ascertained using Wien’s [101] method: existence of corresponding but incongruent clades with bootstrap support greater than 70% are seen as support for not concatenating data sets.

### Test for recombination, network analyses

The Phi Test [43] implemented in Splitstree4 [102] was used to test for recombination in each gene fragment.

The best single gene tree from each locus was combined into a file from which a consensus network was constructed in Splitstree4 [102]. Thresholds used were 0.04 (all splits present in at least one tree, 1/26), 0.08 (splits present in at least two of the trees), 0.5 (splits present in half the trees), 0.9 (splits present in 90% of the trees).

NeighborNet with uncorrected P distance in SplitsTree4 [102,103] was used to visualize conflict in the 273-OTU matrix with 27 concatenated gene fragments.

### Calculation of divergence time

The geologic time scale of Gradstein et al. [104] was used in this study. The term ‘Tertiary’ was replaced by Paleogene and Neogene [105].

Bayesian (BA) estimates in the BEAST V1.7.4 [95,106] software were used to estimate divergence dates using Markov Chain Monte Carlo (MCMC) sampling. Trees were visualized in Figtree V1.3.1 [107]. Many preliminary runs were conducted on the partitioned and unpartitioned data file to explore parameters. Operators were optimized automatically. The final .xml files (Additional files 22, 23) were run ten times using the maximum time available at the Computational Biology Service Unit (CBSU) BioHPC computer cluster at Cornell University. The conditions for the partitioned runs were: 27 unlinked partitions with individual substitution models, estimated frequencies of nucleotides, a random starting tree, an uncorrelated relaxed clock with log normally distributed uncorrelated rates between branches [108], Yule model of speciation (a pure birth process), default operators modified based on preliminary runs, auto-optimization turned on, and parameters were sampled every 10,000 steps. The conditions for the unpartitioned runs were identical except that there was only one partition and one substitution model. Marker-specific and whole-dataset-specific substitution models were determined in Findmodel [109] and are listed in Additional file 2. Findmodel uses Weighbor[110], PAML[111] and methods in Modeltest [112] to determine substitution models. Following the reasoning of Nie et al. [34] a normal prior was used with a mean of 58.5 Ma (st. dev. = 5 Ma) for the stem age of *Vitis.* Liu et al.’s [35] reasoning was adopted to 1) place the second calibration point of *V. labrusca* and closely related North American relatives in the subg. *Vitis* at 5.75 Ma (st. dev. = 0.5 Ma), and 2) fix the stem age of *Vitaceae* with a normal prior distribution of 90.7 Ma (st. dev. = 1 Ma). Runs from the same .xml file were combined if they shared the same trace, met general quality requirements outlined in Additional file 24, and if the addition increased the ESS of key parameters. The three unpartitioned runs with the highest (almost identical) likelihoods were combined after 10% burnin was removed to produce the chronogram in Figure 3. Unpartitioned runs were used because the combined partitioned runs did not have sufficient support for important parameters. Five runs of the partitioned dataset were combined after removing a burnin of 10-75% to compute the evolutionary rates (gene.meanRate) of the individual genes. These gene.meanRates had large ESS. Means and 95% Highest Posterior Density (HPD) from these combined runs were computed using TreeAnnotator.

### Phylogenetic and ancestral area analyses

To evaluate species we used a phylogenetically based general lineage concept, where species are defined as separately evolving segments of metapopulation lineages [80]. Additional criteria of more stringent species definitions were considered, such as monophyly [113] and diagnosability [81,114].

#### Maximum likelihood

The 273-OTU matrix was analyzed under the ML criterion using RAxML [115] versions 7.0.4, 7.2.6 and HPC2 at the web portal of the Cyber Infrastructure for Phylogenetic Research (CIPRES) cluster. The data were partitioned by gene fragment (Additional file 21). All characters were included. Gap coding was removed. Indels were treated as missing data. Twenty replicate searches were run on this final data set using a rapid hill climbing algorithm and the GTRGAMMA (= GTR + Optimization of substitution rates + GAMMA model of rate heterogeneity, the alpha parameter was estimated) model of substitution as recommended by the program’s author, and the default of 25 rate categories. The rapid bootstrapping option [116] was chosen to generate 1,000 bootstrap replicates. The best-scoring ML tree was obtained in the same search and bootstrap values were annotated. Output was visualized in Dendroscope V2.2.2 [117].

To test for conflict among genes, a preliminary analysis was performed for each gene fragment using the same parameters as above but without a partitioned model. Each gene was run in 10 replicates. Five more replicates were added if the final maximum likelihood values varied extensively among replicates. To keep trees comparable no OTUs were excluded. One-thousand bootstrap replicates were collected for each gene fragment. Incongruent clades with bootstrap support of 70% or greater were considered as support for not combining data sets [42,100]. Due to computational limitations, the single gene analyses were performed only in RAxML at CIPRES. Because of the sparse yield of information and low information content of most markers, this computational expense was not repeated with the 273-OTU matrix.

#### Bayesian (BA)

Bayesian analyses were performed on the 273-OTU matrix using Mr. Bayes [118] on the concatenated, non-partitioned data set using the K80 substitution model (Nst = 2, 4 by 4) plus Gamma, as determined in Findmodel. Multiple short runs were performed to determine the temperature and number of chains that would support chain swap. The two final and longest runs of 48 and 50 million generations (fitting just within the 168 hr time limit) were run with 8 chains, temperature of 0.10, and sample frequency every 5,000 generations. Tracer V1.5 [119] was used to evaluate the MCMC runs, TreeAnnotator v1.6.1 [95] and Figtree v1.3.1 were used to annotate and visualize maximum credibility trees listing all posterior probabilities. Burn in was 2.5 million (50 mil run) and 10 million (48 mil run).

#### Maximum Parsimony (MP)

The software package TNT, Hennig Society version [120] was used to analyze the unpartitioned 273-OTU matrix under parsimony, assuming unordered character state transformation and equal weights (Fitch parsimony) [121]. Uninformative characters were excluded and gaps were coded. The efficient option of “driven search” in TNT was used for the search. This option searched until a minimum tree length was found a certain number of times and then a consensus was estimated. After a second round of searching the new consensus was compared to the previous one, and so on until the consensus stabilized [122]. The driven searches included the ratchet [123], tree drifting, tree fusion and sectorial search [124]. The default settings were used except that the consensus was stabilized four times instead of twice. The search was repeated four times using a different random starting seed and without specifying a target score. The strict consensus tree was constructed using all most parsimonious trees from all four searches. Bootstrap support was based on 1,000 replicates of driven searches using the same search components and default parameters. Synapomorphies were optimized and listed, and those of interest were reconstructed. The character values, indicating the level of homoplasy, were obtained in TNT to study the support at nodes of interest. Trees for illustrations were exported in a NEXUS format, manually converted to Newick trees, visualized and annotated using Dendroscope V 2.2.2.

#### Ancestral area analysis

The geographic distribution was partitioned into four continental area units that correspond to broad distributional trends in *Vitis*: Asia, Europe/near East, E- and SE-North America (including Mexico and the Caribbean) and Western North America (Figure 1). An area code was added to each accession in the 273-OTU data matrix (Additional file 20). Fitch optimization (reversible parsimony) [121] was performed in TNT to optimize the area on the strict consensus tree [125].

## Appendix 1

### Biogeography: the Eastern Asia-Eastern North American disjunction

The genus *Vitis* contributes to one of the great distributional phenomena in plant biogeography, the Eastern Asia-Eastern North American disjunction among the temperate to warm temperate northern hemisphere taxa [69,126-129]. Up to 30 species are native to a vast area in eastern Asia, China, Japan and Java, two species across middle Asia and Europe, and up to 28 species across the eastern and southwestern US and Mexico [2] (Figure 1). A small number of species extend into the Tropics both in Asia and in North America [6,130-132]. There is widespread agreement that these disjunct floras are relicts of plant communities that were distributed throughout a large part of the Northern Hemisphere during much of the Paleogene and early Neogene (formerly the Tertiary) Periods (65-15 Ma) [69,70,126,129,133,134]. Communities on different continents were linked by migration across the Bering Land Bridge, linking North America and Asia beginning in the Miocene [133], and across the North Atlantic Land Bridge, linking North America and Europe particularly in the early Eocene [65,129]. Wild grapes are a savored food of birds and some small mammals, providing dispersal for these species. Intra-continental migration was impeded between Europe and Asia by an epicontinental seaway (Cretaceous-Eocene) as was migration between east and west North America (upper Cretaceous), followed by regions of dry continental climates [65,129,134]. Climatic cooling at the start of the Oligocene (33.9 Ma) gave rise to the Mixed Mesophytic Forest of deciduous and evergreen trees and associated taxa that comprise the modern Paleogene/Neogene relict floras [134], among them the early grapevines. The flora retreated into refugial regions in response to Pliocene cooling (5.3-2.5 Ma) and Quaternary glaciations (2.5-0 Ma) [134]. Tectonic uplifting of mountain ranges and plateaus during the Pliocene into the Holocene, and concurrent reduction in precipitation caused further partitioning of the East Asian habitats [63,133]. Fossil distributions suggest that, by the end of the Neogene, the genus*Vitis* was widely distributed in the Northern Hemisphere [10]. As detailed in Nei et al. [34], the oldest reliable *Vitis* seeds are from the Paleocene (65.5-55.8 Ma) [53,54] and were not detected in the preceding Cretaceous period. Important estimated time points in the Vitaceae diversifications were: 1) the divergence of Vitaceae and Leeaceae (stem age of Vitaceae), estimated by Magallón and Castillo [57] at 90.82 – 90.65 Ma, this estimate was based on a five gene data set (chloroplast rbcL, atpB,*matK*,and nuclear 18S and 26S nrDNA) obtained from GenBank, and conversion to absolute time using three fossil reference time points, 2) the divergence of the *Ampelocissus*-*Vitis* clade in the Tiffian stage of the Paleocene (62.0-56.8 Ma) based on fossil evidence synthesized in Nei at al. [34], and 3) the presence of well preserved *Vitis* seed at the late Neogene Gray Fossil site in Tennessee (7-4.5 Ma) [59].

### Supporting data

The data sets supporting the results of this article are included within the article and its additional files. NCBI accession numbers: [Genbank: JX952227-JX960379, EMBL: HF544510-HF544512]. Additional file 1 lists the sequence accession number for all OTUs; Additional file 2 lists accession numbers by marker. Alignment, phylogenies, trees and BEAST .xml files were deposited at http://dx.doi.org/10.5061/dryad.s1s75.

## Competing interests

The authors declare they have no competing interests.

## Authors’ contributions

YW, CJS, AMB, HRS conceived the ideas; YW, CJS collected or arranged for the materials; AMB, YW, JAL developed the markers; YW, AMB developed the sequence matrix; HRS analyzed the data except for PCA; AMB developed and performed PCA; HRS, JAL, YW wrote the paper; CJS and GYZ managed the project. All authors read and approved the final submission.

## Supplementary Material

Additional file 1 **Accession Information Table_with GenBankAccNo.xlsx.** Germplasm accessions information. Accession name, taxonomy, continental origin, source, data set membership, individual GenBank and EMBL accession numbers. Click here for file

Additional file 2 **Fragment information.xlsx.** Gene fragment information. Primer sequence, fragment length, chromosome location, original EST annotation, number of unique site patterns (an indicator of informative variation), gene.meanRate, substitution models used (from Findmodel), GenBank accession numbers. Click here for file

Additional file 3 **a-d. Consensus Networks.pdf.** Consensus Network of 26 single gene trees, showing all splits present in at least **a.** one tree (1/26, threshold = 0.04), **b.** two trees (2/26, threshold = 0.08), **c.** 50% of the trees (threshold = 0.5), **d.** 90% of the trees (threshold = 0.9). Click here for file

Additional file 4 **Node Ages.pdf.** Node ages (Ma) of all nodes in maximum clade credibility tree inferred with BEAST from three combined runs. Click here for file

Additional file 5 **Posterior Probabilities.pdf.** Posterior probabilities of all nodes in maximum clade credibility tree inferred with BEAST from three combined runs. Click here for file

Additional file 6 **Cartoon BestMLtree.pdf.** Cartoon of best ML tree. For comparison with trees reconstructed with other methods. Blue = North American OTUs, Green = Asian OTUs, Red = European (mostly) OTUs. Click here for file

Additional file 7 **Best ML tree with Bootstrap supports.pdf.** Best ML tree of 273 accessions with bootstrap supports from 1,000 replicates. Supports 1-100% are listed along branches. Abbreviated uncorrected taxon labels. Click here for file

Additional file 8 **Bayesian Tree_48MGen.pdf.** Bayesian tree, 48 million generations, not partitioned, burn in 10 million steps. Posterior probabilities (0 to 1) are listed along branches. Click here for file

Additional file 9 **BayesianTree_50MilGen.pdf.** Bayesian tree, 50 million generations, not partitioned, burn in 5%. Posterior probabilities (0 to 1) are listed along branches. Click here for file

Additional file 10 **Cartoon Of Bayesian Tree_48MilGen.pdf.** Cartoon of Bayesian tree 48 million generations, not partitioned, burn in 10 million steps. Click here for file

Additional file 11 **Nodes With Support And Character scores Genes Filled.xlsx.** List of characters supporting selected nodes in strict consensus tree (Figure 3), their degree of homoplasy and marker identity. A character value of one indicates that the character changed once in the phylogeny and there is no homoplasy. A character value of 10 means that the character changed 10 times in the phylogeny. Click here for file

Additional file 12 **Node Numbers of MP strict consensus tree.pdf.** Node numbers of MP strict consensus tree (Figure 4A-B), correspond to node numbers in Additional file 11. Click here for file

Additional file 13 **MP BS tree 1000rep.pdf.** Maximum parsimony Bootstrap tree, 1000 replicates. Abbreviated taxon labels. Support values >50% are indicated. Click here for file

Additional file 14 **Ancestral Area optimization_JacquIsAsian.pdf.** Ancestral Area Fitch Parsimony optimization on strict consensus tree. Green = Eastern/Southeastern North America including Mexico; Yellow = Western North America; Red = Asia; Blue = Europe/Near East. Click here for file

Additional file 15 **Vitis classifications.pdf.** Classifications of *Vitis* proposed by six major systematists between 1895 and 1991. Click here for file

Additional file 16 **MP Branch lengths.pdf.** Branch lengths for the strict consensus tree of the MP driven search. Abbreviated unmodified taxon labels. Branch length reflects the number of character changes along a specific branch. Click here for file

Additional file 17 **Laboratory procedures.pdf.** PCR and cycle sequencing protocols. Click here for file

Additional file 18 **PCA scatter plot.pdf.** Pink dots represent Asian species, green and blue dots North American species, brown dots European species, black dots intercontinental hybrids. The numbers associated with the dots correspond to accession numbers in Additional file 1. Circled accessions are discussed in text. Click here for file

Additional file 19 **TNTfile_303OTU_WithSIC_uninformCharsDeact.tnt.** TNT data file including all 303 original accessions, gap coded. This file was used for some preliminary analyses and can be opened with a text editor. Click here for file

Additional file 20 **TNTinfile_273otu_noAmpWith272_6670fixed_sic_LastAreaChar_JacquIsAsian.tnt.** The .TNT file used in the present study (273 OTU, simple indel coding, in TNT format). Last character in each sequence was added for the ancestral area reconstruction. Can be opened using a text editor. Gene partitions are listed in Additional file 21. Click here for file

Additional file 21 Gene partitions for the 11437bp matrix. Click here for file

Additional file 22 **800mil10Klog_52taxaNoTiliWithCay_11437_LognRelYule3CalPts_NOpartK80G.xml.** XML file used to date divergences in BEAST (unpartitioned). Click here for file

Additional file 23 **400Mil10Klog_53taxa With Cayratia_11437_Yule_LogNrelaxedClock_partitioned3calibration Many Models.xml.** XML file used to estimate evolutionary rate of change per partition (27 partitions). Click here for file

Additional file 24 **Evaluation criteria for BEAST runs.pdf.** Evaluation criteria for Tracer files. Click here for file
