# Supplementary material for: A phylogenetic analysis of the grape genus (Vitis L.) reveals broad reticulation and concurrent diversification during neogene and quaternary climate change
Source: BMC Evol Biol. 2013 Jul 5;13:141. doi: 10.1186/1471-2148-13-141 (PMC3750556; doi:10.1186/1471-2148-13-141)

Additional File 3a.

Consensus Network of 26 gene trees showing all splits found in at least one tree (1/26, 0.04).

Abbreviated, original taxa names.

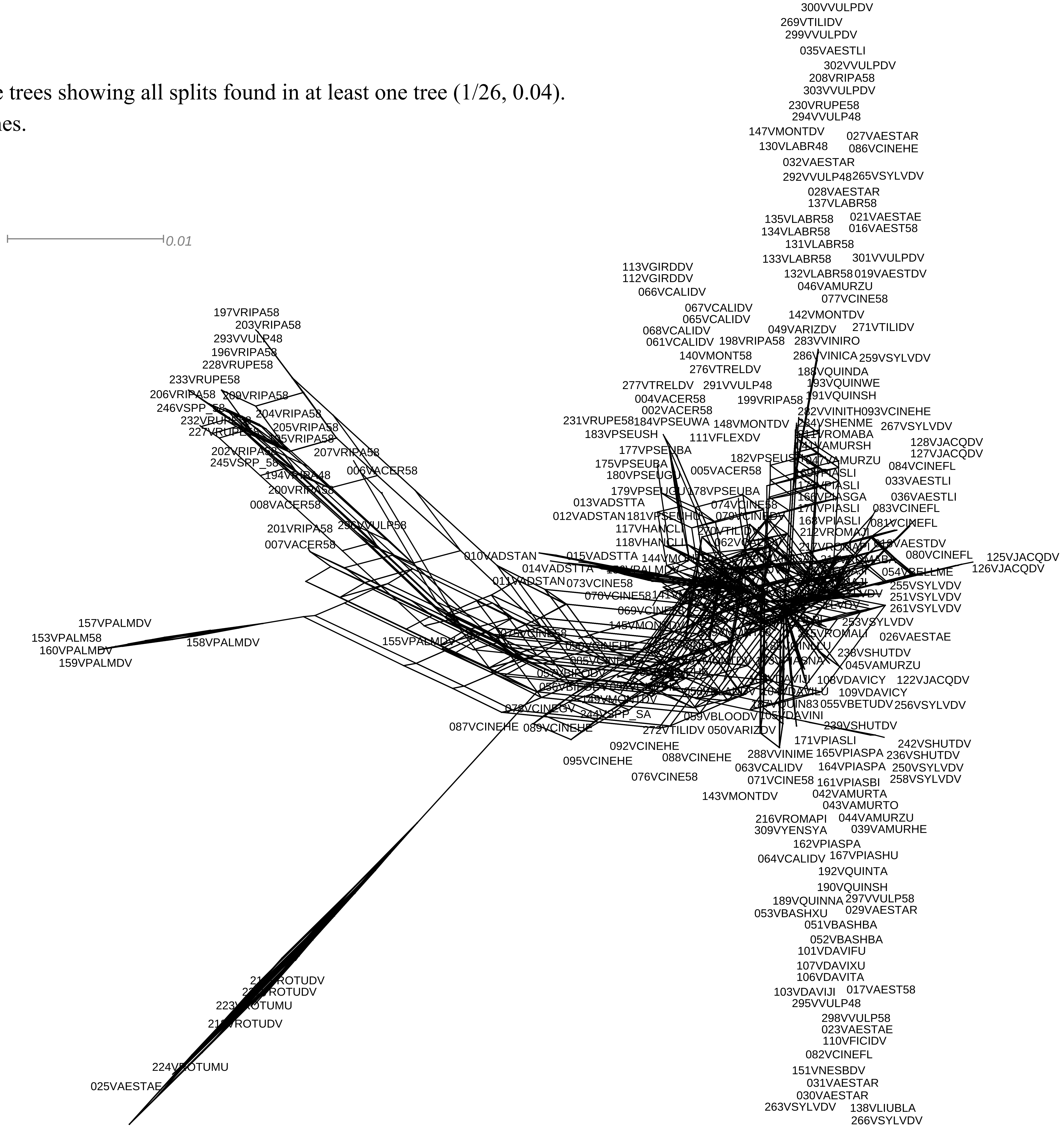

Additional File 3b.
Consensus Network of 26 gene trees showing all splits found in at least two tree (1/26, 0.08). Abbreviated original taxa names.

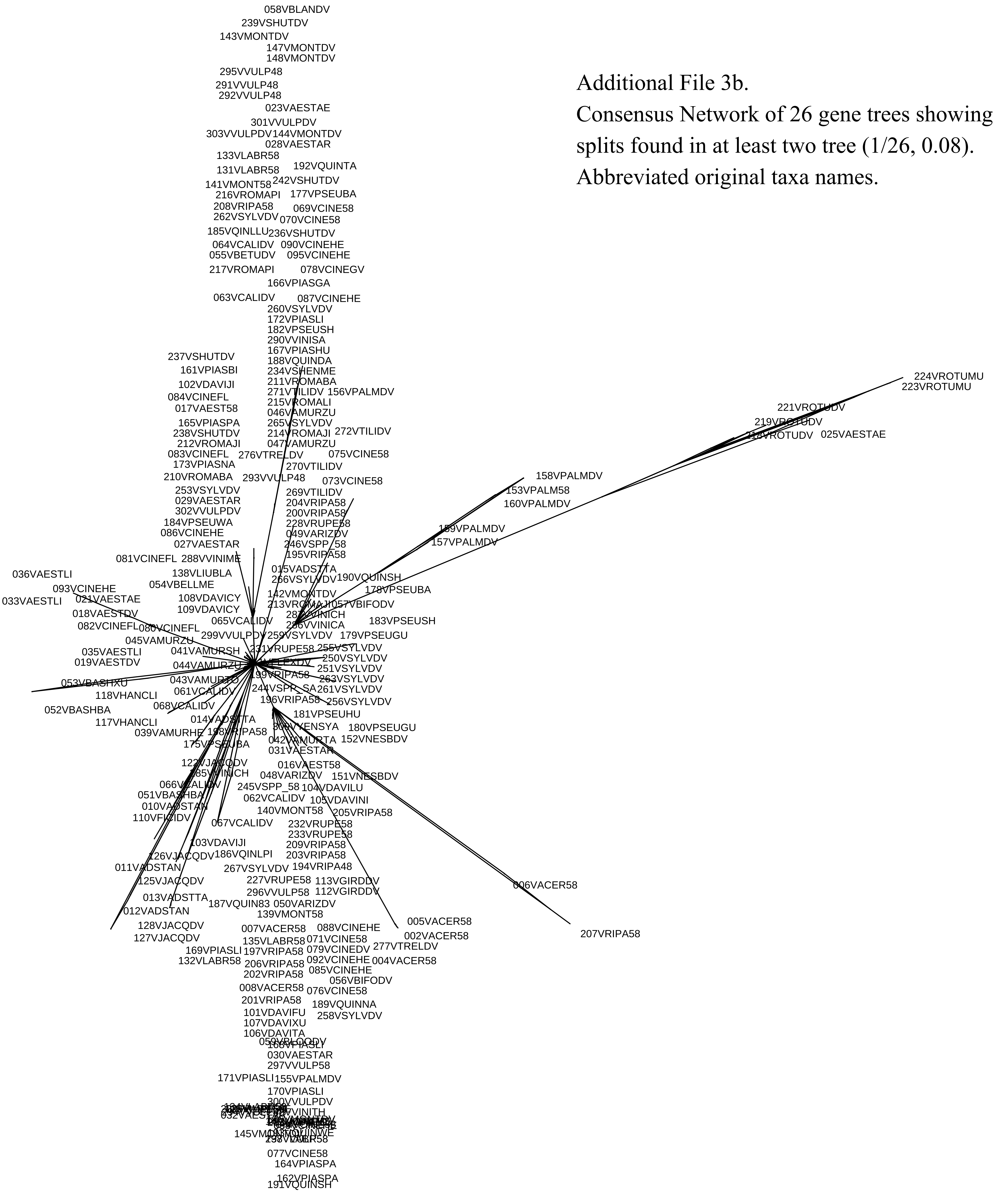

Additional File 3c.

Consensus Network of 26 gene trees showing all splits found in at least 50% of gene  tree (13/26, 0.5). Abbreviated original taxa names.

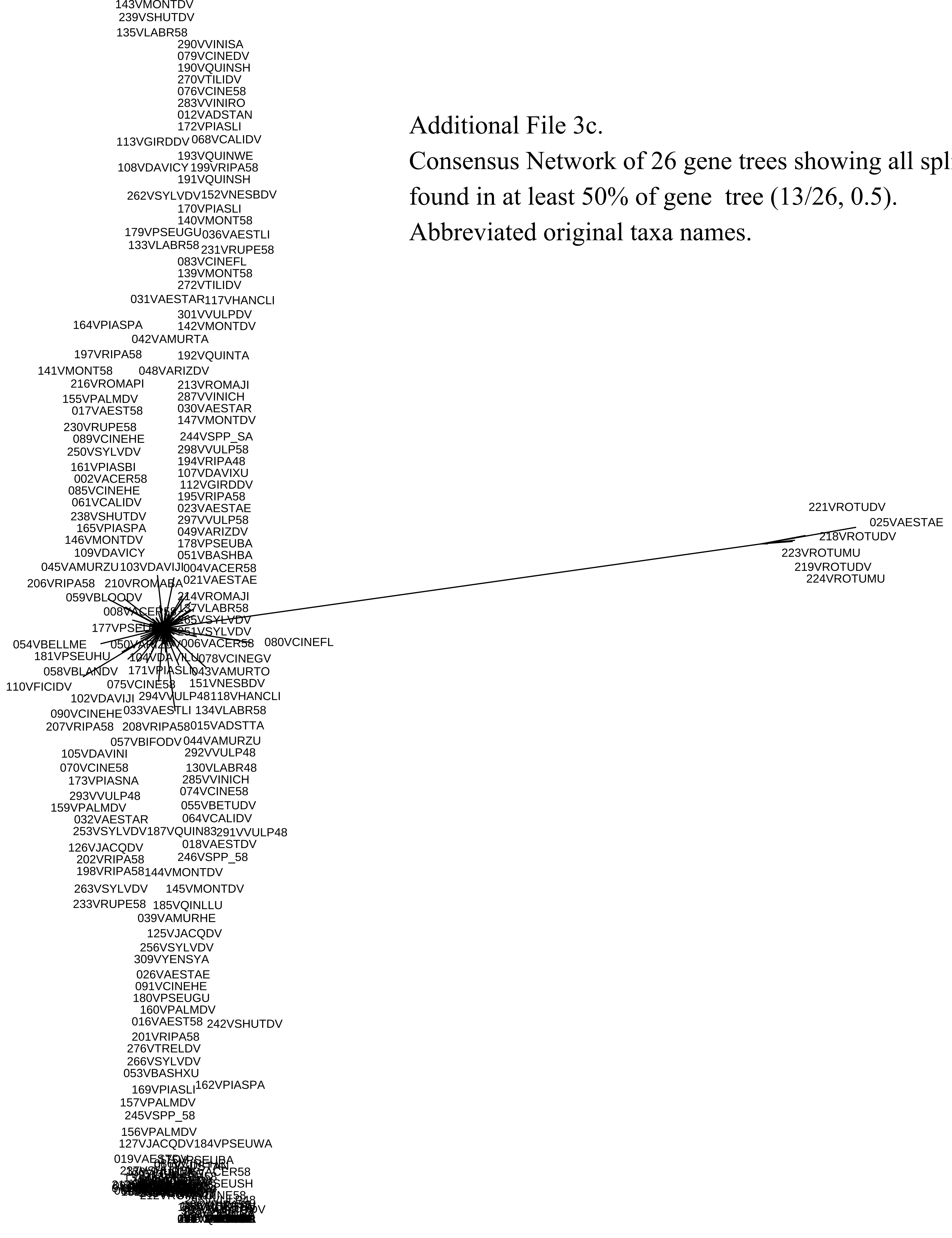

Additional File 3d.

Consensus Network of 26 gene trees showing all splits found in at least 90% of gene  tree (23.4/26, 0.9).

Abbreviated original taxa names.

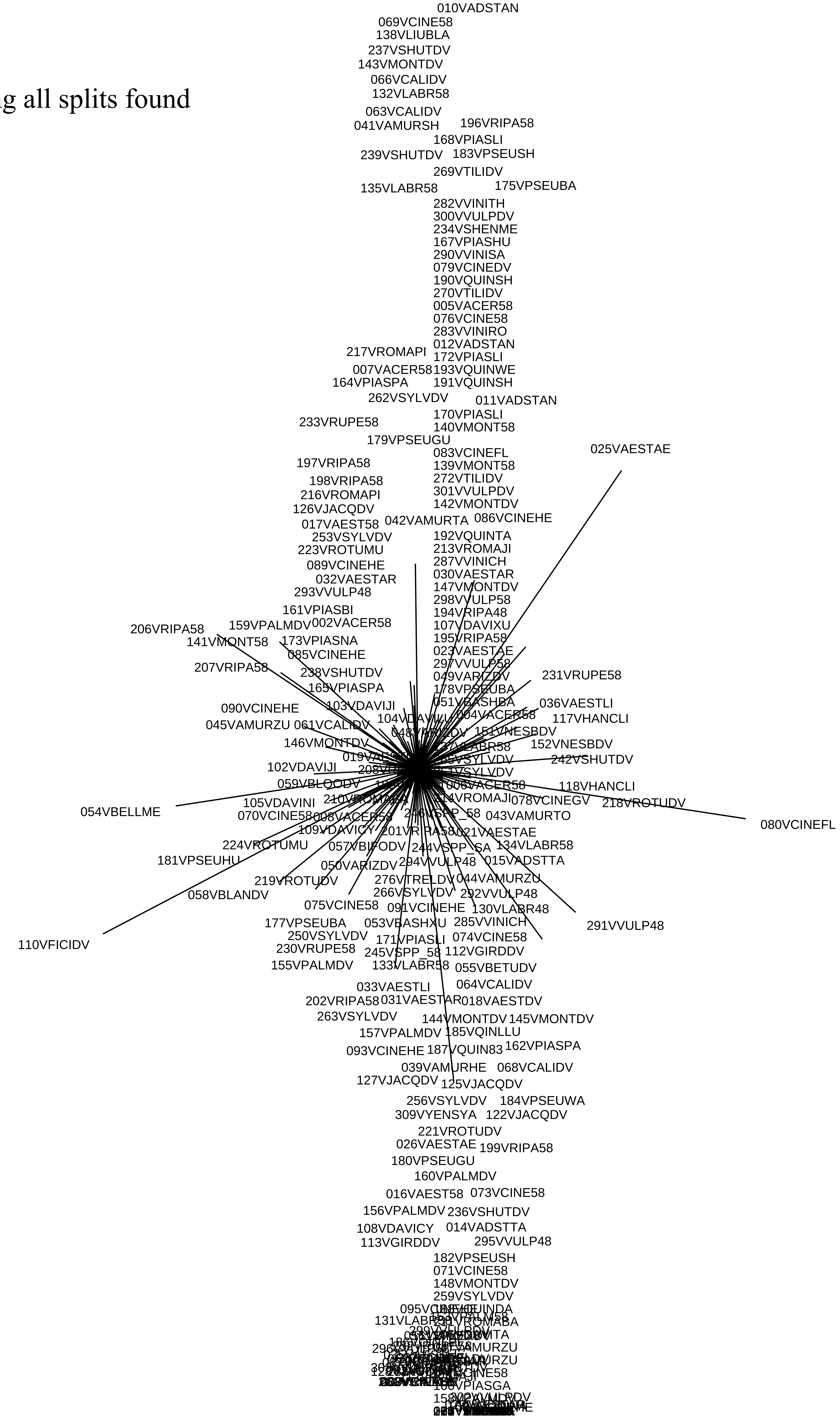

Supplement: Additional file 3 — a-d. Consensus Networks.pdf. Consensus Network of 26 single gene trees, showing all splits present in at least a. one tree (1/26, threshold = 0.04), b. two trees (2/26, threshold = 0.08), c. 50% of the trees (threshold = 0.5), d. 90% of the trees (threshold = 0.9). [file 1471-2148-13-141-S3.pdf]
