# Supplementary figures and images for: A phylogenetic analysis of the grape genus (Vitis L.) reveals broad reticulation and concurrent diversification during neogene and quaternary climate change
Source: BMC Evol Biol. 2013 Jul 5;13:141. doi: 10.1186/1471-2148-13-141 (PMC3750556; doi:10.1186/1471-2148-13-141)

## Additional File 4. Node Ages (Ma)

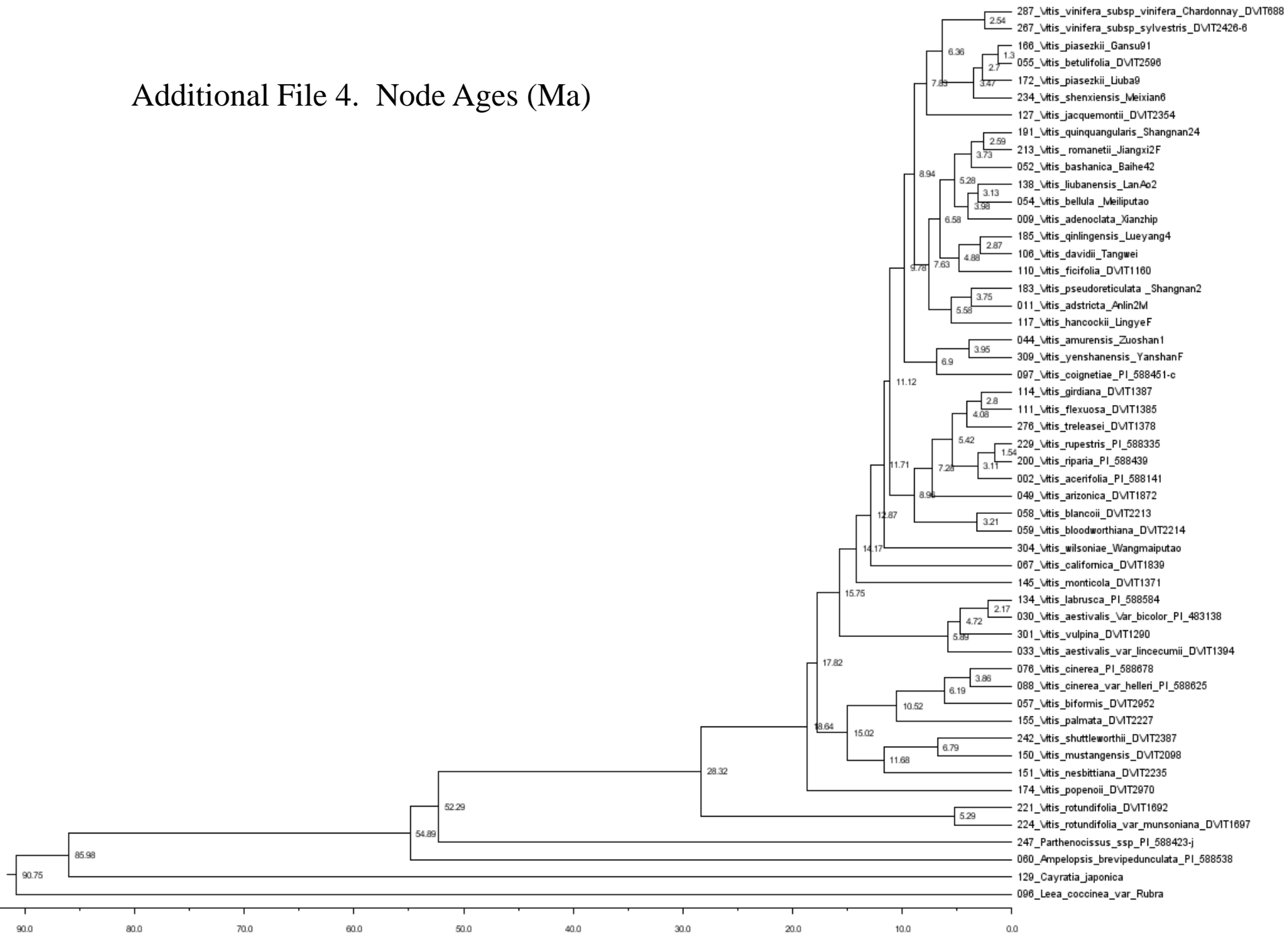

Supplement: Additional file 4 — Node Ages.pdf. Node ages (Ma) of all nodes in maximum clade credibility tree inferred with BEAST from three combined runs. [file 1471-2148-13-141-S4.pdf]

Additional File 5:  
Posterior Probabilities

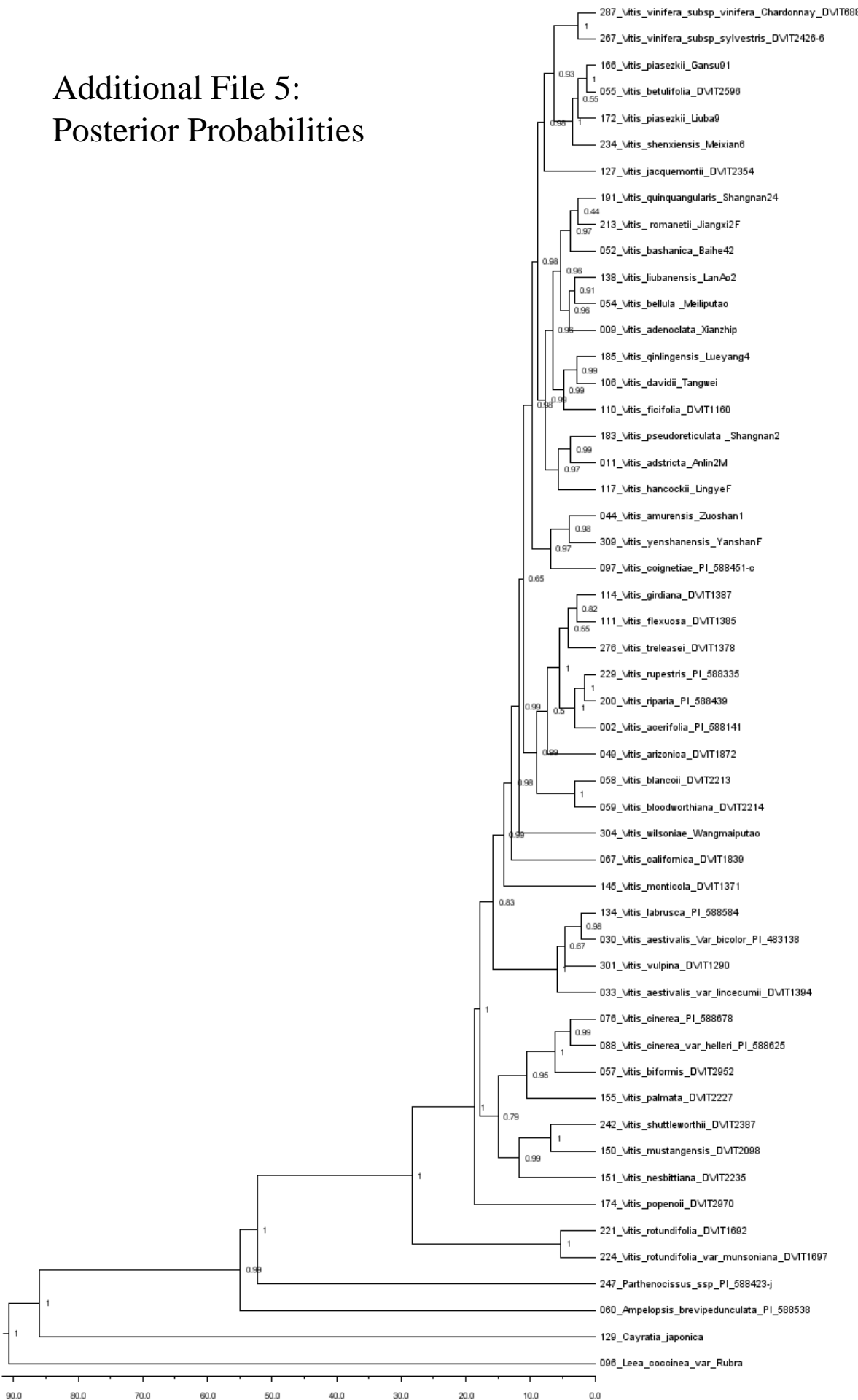

Supplement: Additional file 5 — Posterior Probabilities.pdf. Posterior probabilities of all nodes in maximum clade credibility tree inferred with BEAST from three combined runs. [file 1471-2148-13-141-S5.pdf]

Additional File 6.  
Cartoon of best ML tree

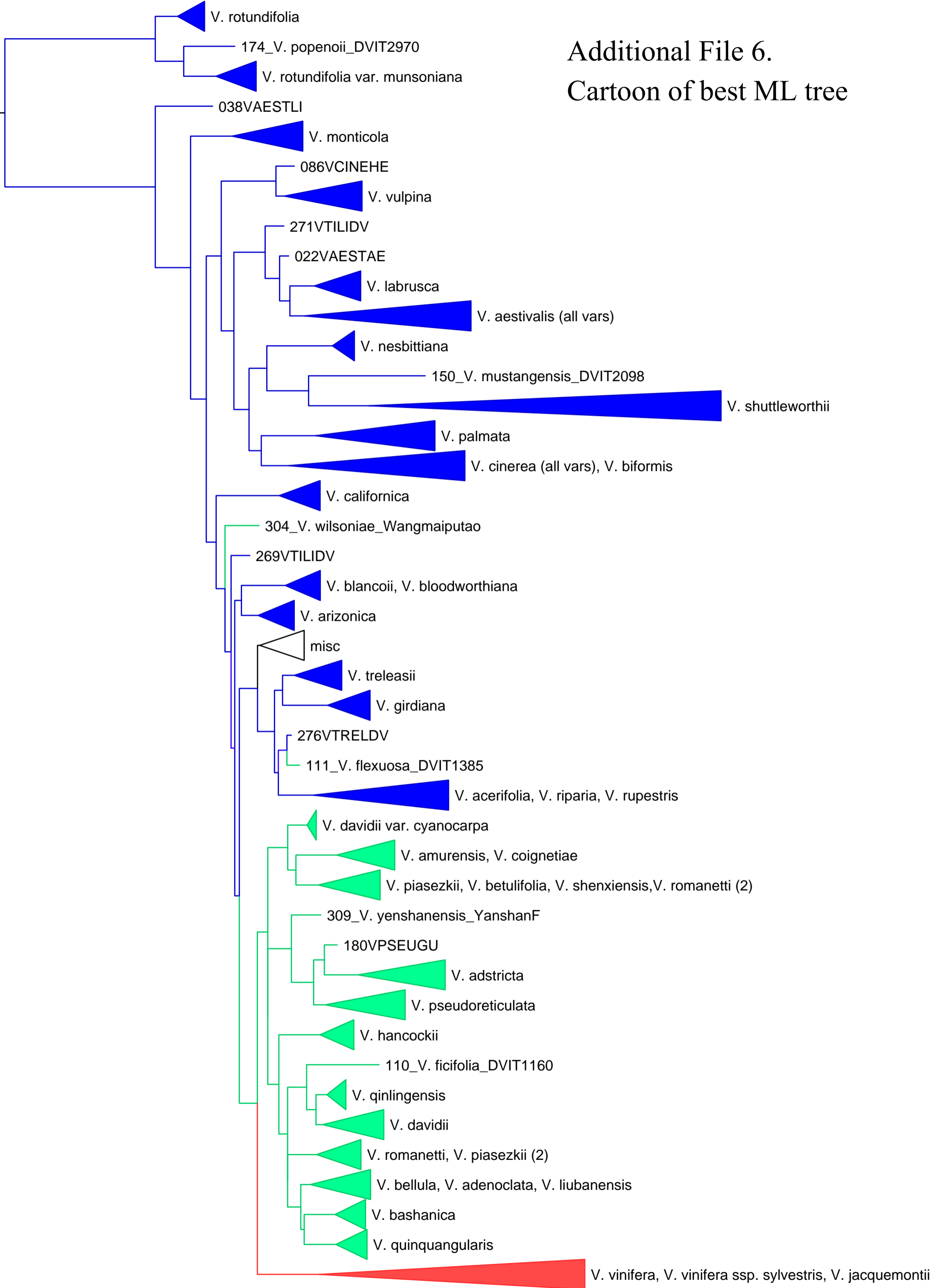

Supplement: Additional file 6 — Cartoon BestMLtree.pdf. Cartoon of best ML tree. For comparison with trees reconstructed with other methods. Blue = North American OTUs, Green = Asian OTUs, Red = European (mostly) OTUs. [file 1471-2148-13-141-S6.pdf]

## Additional File 8. Bayesian Tree

Posterior probabilities (0 to 1) are listed.

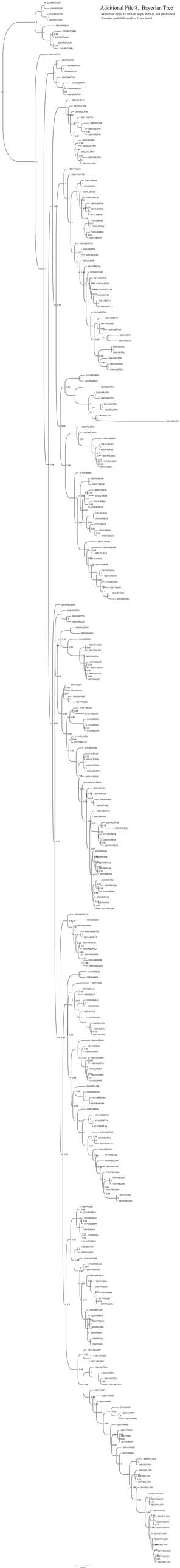

Supplement: Additional file 8 — Bayesian Tree_48MGen.pdf. Bayesian tree, 48 million generations, not partitioned, burn in 10 million steps. Posterior probabilities (0 to 1) are listed along branches. [file 1471-2148-13-141-S8.pdf]

Additional File 10. Cartoon of Bayesian Tree  
48 million steps, not partitioned,  
burn-in 10 million steps.

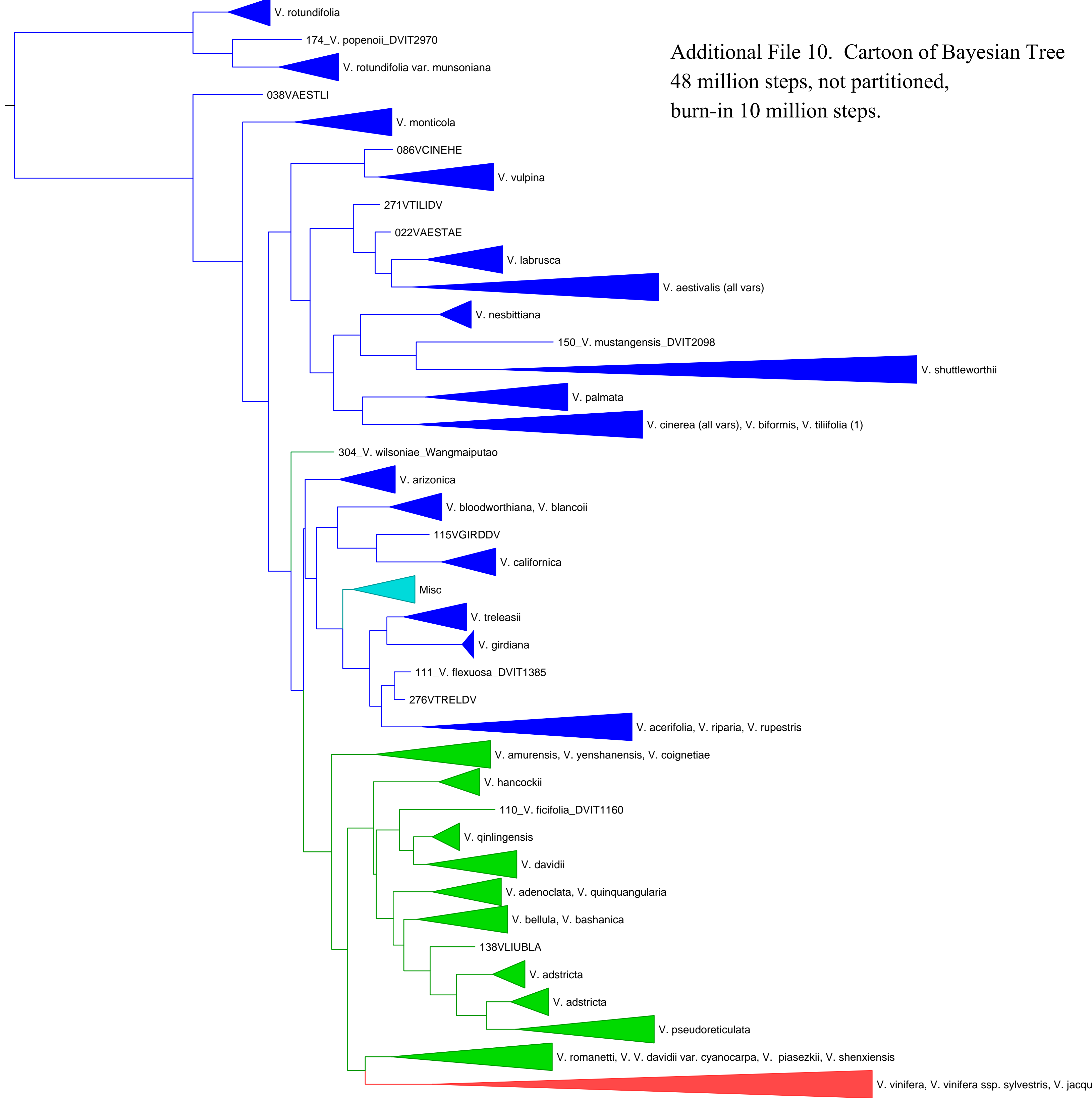

Supplement: Additional file 10 — Cartoon Of Bayesian Tree_48MilGen.pdf. Cartoon of Bayesian tree 48 million generations, not partitioned, burn in 10 million steps. [file 1471-2148-13-141-S10.pdf]

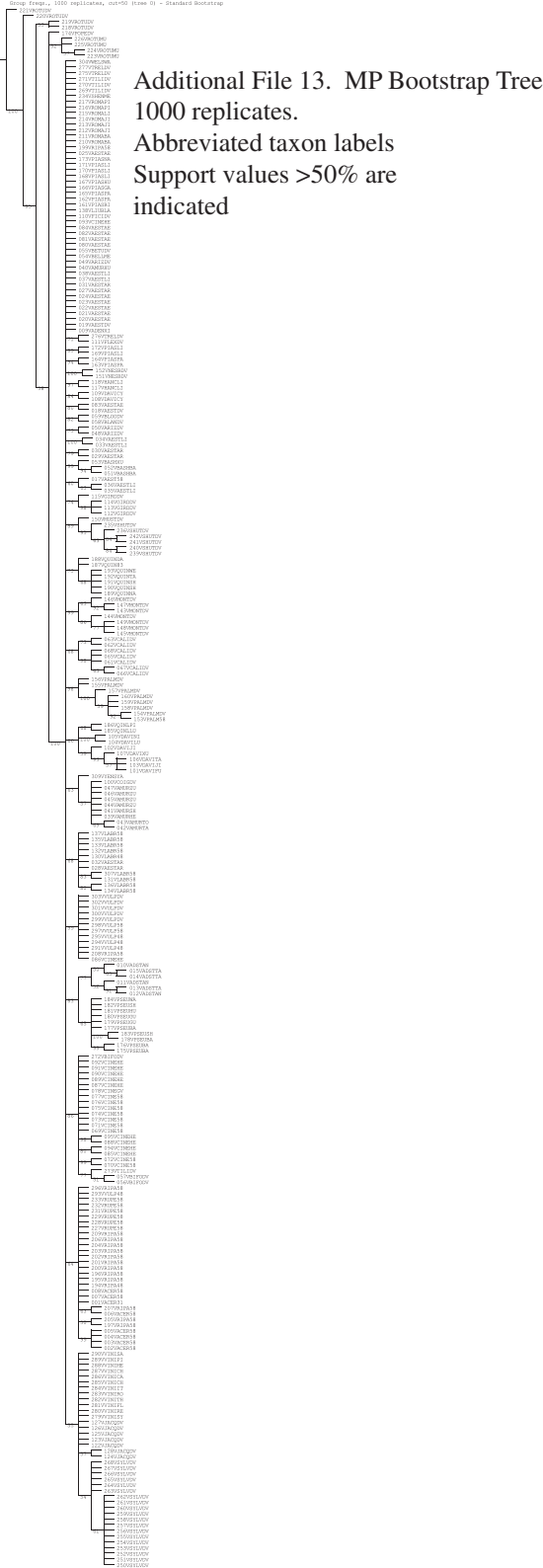

Supplement: Additional file 13 — MP BS tree 1000rep.pdf. Maximum parsimony Bootstrap tree, 1000 replicates. Abbreviated taxon labels. Support values >50% are indicated. [file 1471-2148-13-141-S13.pdf]

of character changes along a specific branch.

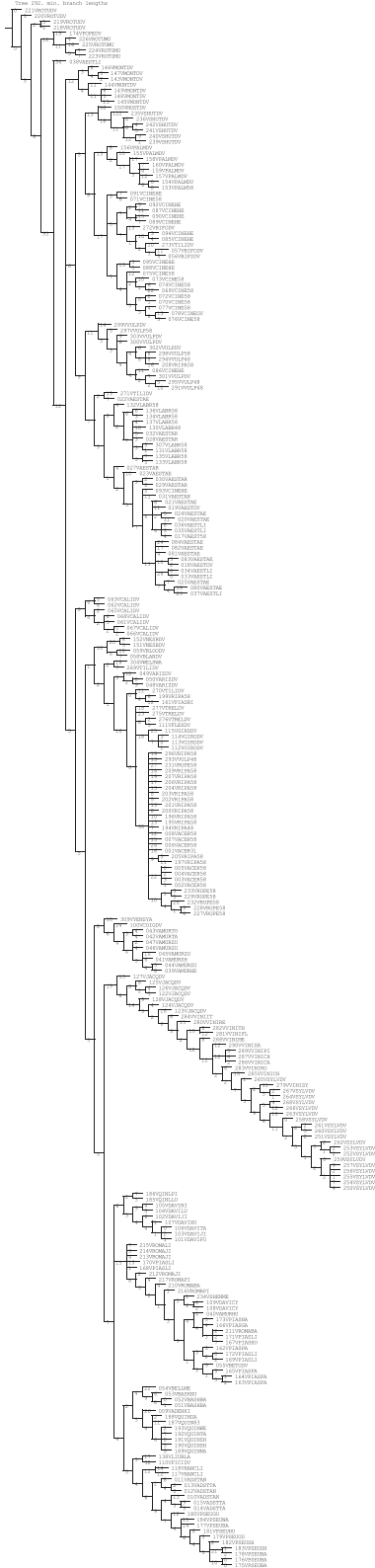

Supplement: Additional file 16 — MP Branch lengths.pdf. Branch lengths for the strict consensus tree of the MP driven search. Abbreviated unmodified taxon labels. Branch length reflects the number of character changes along a specific branch. [file 1471-2148-13-141-S16.pdf]

# Additional File 18: PCA scatter plot

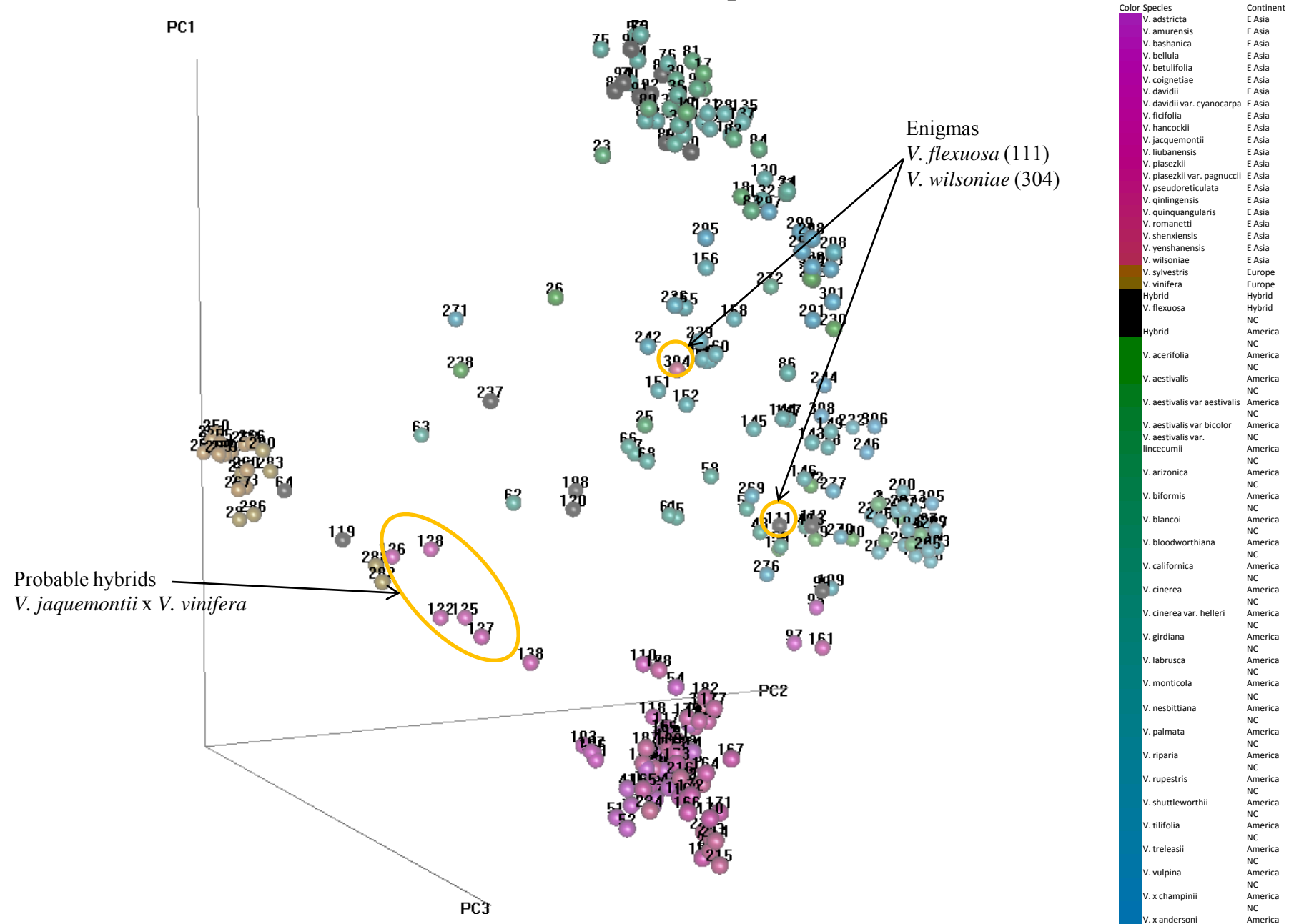

Supplement: Additional file 18 — PCA scatter plot.pdf. Pink dots represent Asian species, green and blue dots North American species, brown dots European species, black dots intercontinental hybrids. The numbers associated with the dots correspond to accession numbers in Additional file 1. Circled accessions are discussed in text. [file 1471-2148-13-141-S18.pdf]
