# Supplementary material for: A phylogenetic analysis of the grape genus (Vitis L.) reveals broad reticulation and concurrent diversification during neogene and quaternary climate change
Source: BMC Evol Biol. 2013 Jul 5;13:141. doi: 10.1186/1471-2148-13-141 (PMC3750556; doi:10.1186/1471-2148-13-141)

Additional File 7. ML bootstrap supports on best scoring tree.
Best ML tree of 273 accessions with bootstrap supports from 1,000 replicates.
Supports 1-100% are listed along branches. Abbreviated original taxon labels.

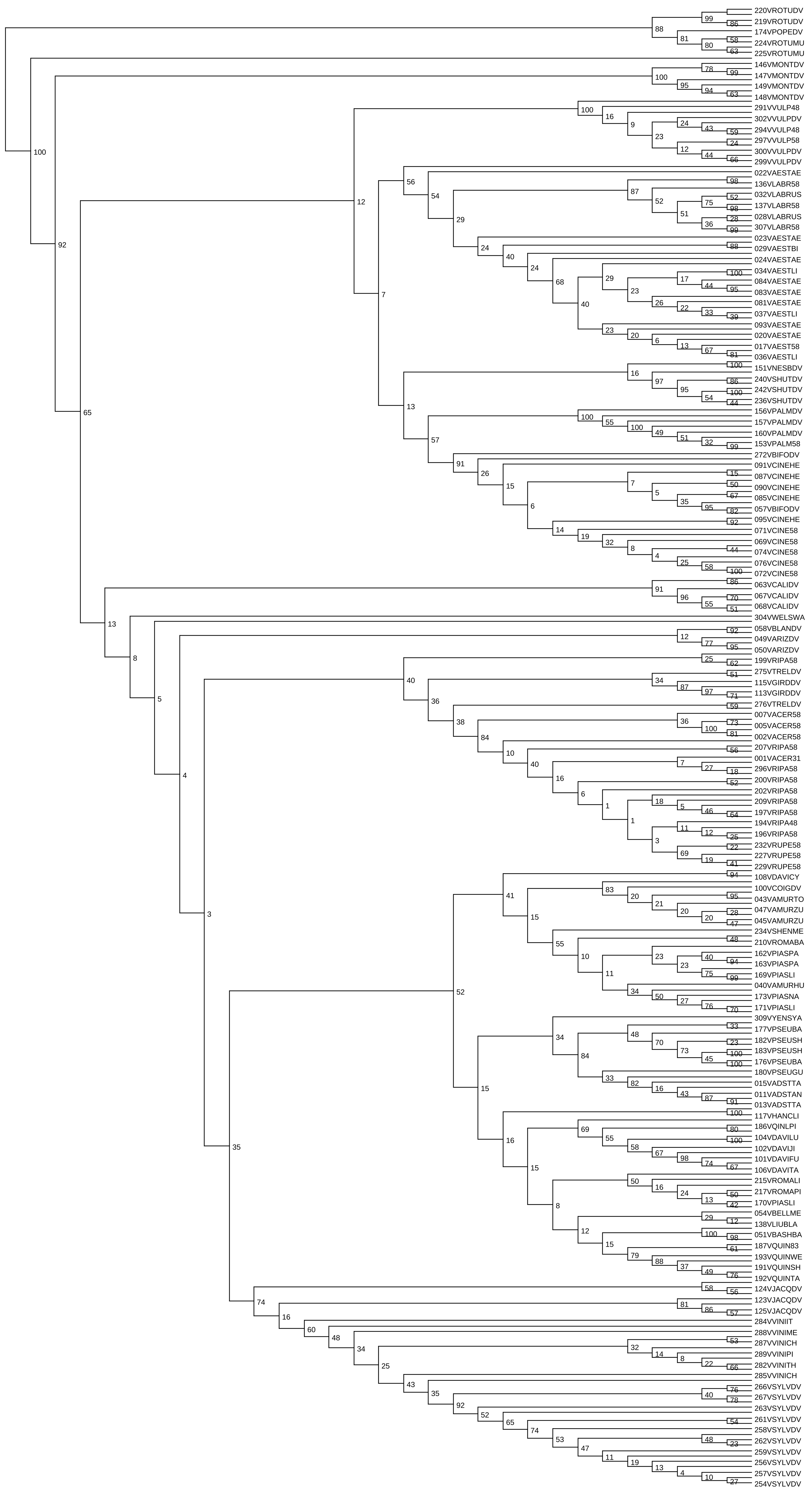

Supplement: Additional file 7 — Best ML tree with Bootstrap supports.pdf. Best ML tree of 273 accessions with bootstrap supports from 1,000 replicates. Supports 1-100% are listed along branches. Abbreviated uncorrected taxon labels. [file 1471-2148-13-141-S7.pdf]
