# Supplementary material for: A phylogenetic analysis of the grape genus (Vitis L.) reveals broad reticulation and concurrent diversification during neogene and quaternary climate change
Source: BMC Evol Biol. 2013 Jul 5;13:141. doi: 10.1186/1471-2148-13-141 (PMC3750556; doi:10.1186/1471-2148-13-141)

Additional File 9. Bayesian Tree

50 million steps, not partitioned, 5% burn-in. Posterior probabilities (0-1) are listed.

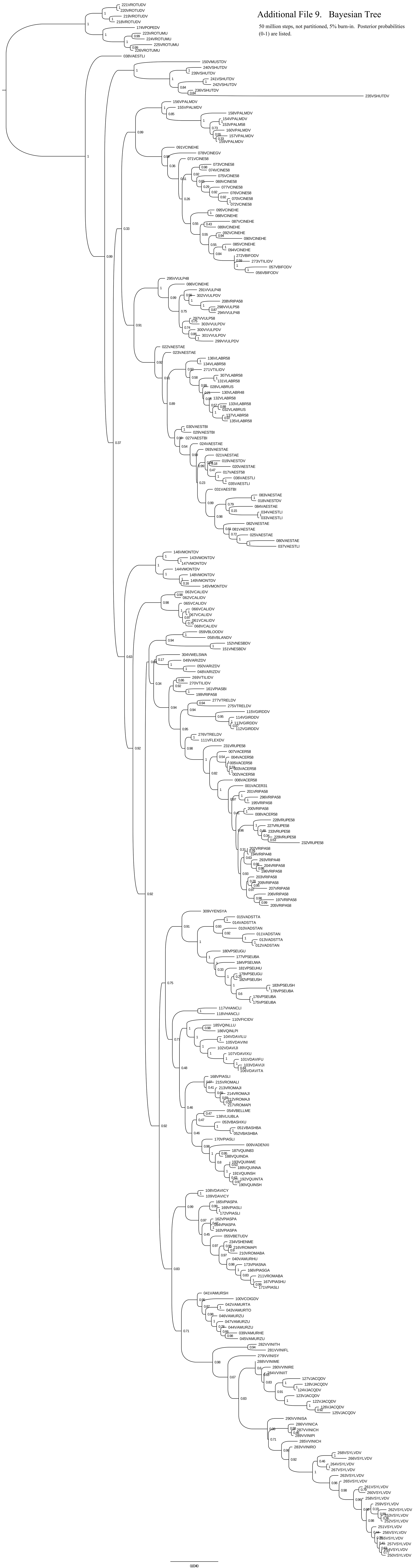

Supplement: Additional file 9 — BayesianTree_50MilGen.pdf. Bayesian tree, 50 million generations, not partitioned, burn in 5%. Posterior probabilities (0 to 1) are listed along branches. [file 1471-2148-13-141-S9.pdf]
