# Supplementary material for: A phylogenetic analysis of the grape genus (Vitis L.) reveals broad reticulation and concurrent diversification during neogene and quaternary climate change
Source: BMC Evol Biol. 2013 Jul 5;13:141. doi: 10.1186/1471-2148-13-141 (PMC3750556; doi:10.1186/1471-2148-13-141)

Additional File 12.

Node numbers of MP strict consensus tree.
Numbers correspond to node numbers
in Additional File 11.

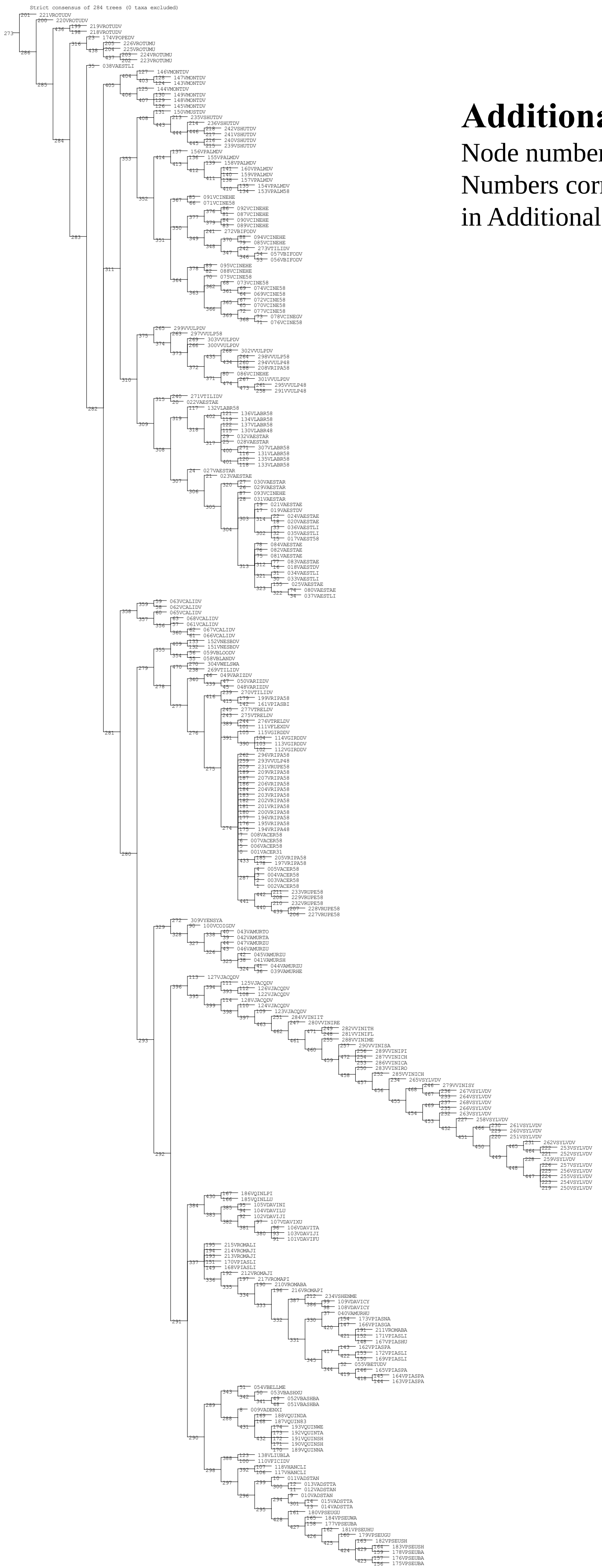

Supplement: Additional file 12 — Node Numbers of MP strict consensus tree.pdf. Node numbers of MP strict consensus tree (Figure 4A-B), correspond to node numbers in Additional file 11. [file 1471-2148-13-141-S12.pdf]
