# Supplementary material for: A phylogenetic analysis of the grape genus (Vitis L.) reveals broad reticulation and concurrent diversification during neogene and quaternary climate change
Source: BMC Evol Biol. 2013 Jul 5;13:141. doi: 10.1186/1471-2148-13-141 (PMC3750556; doi:10.1186/1471-2148-13-141)

Additional File 14.

Ancestral Area Optimization

Areas are identified in Figure 1

Green: Eastern/Southeastern North America including Mexico

Yellow: Western North America

Red: Asia

Blue: Europe/Near East

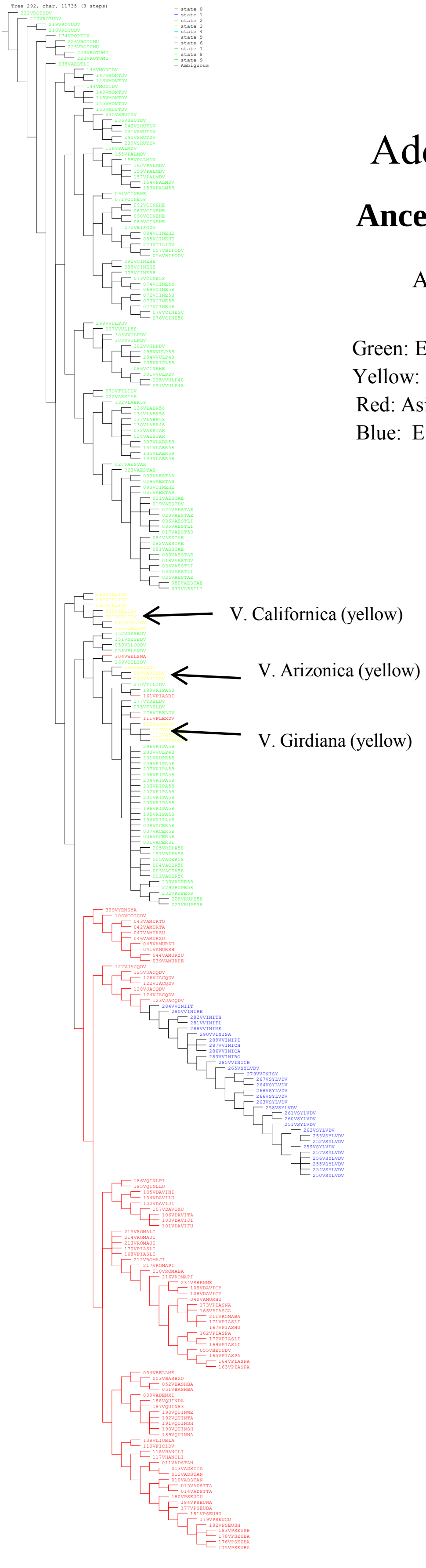

Supplement: Additional file 14 — Ancestral Area optimization_JacquIsAsian.pdf. Ancestral Area Fitch Parsimony optimization on strict consensus tree. Green = Eastern/Southeastern North America including Mexico; Yellow = Western North America; Red = Asia; Blue = Europe/Near East. [file 1471-2148-13-141-S14.pdf]
