# Supplementary material for: A phylogenetic analysis of the grape genus (Vitis L.) reveals broad reticulation and concurrent diversification during neogene and quaternary climate change
Source: BMC Evol Biol. 2013 Jul 5;13:141. doi: 10.1186/1471-2148-13-141 (PMC3750556; doi:10.1186/1471-2148-13-141)
Supplement: Additional file 15 — Vitis classifications.pdf. Classifications of Vitis proposed by six major systematists between 1895 and 1991. [file 1471-2148-13-141-S15.pdf]

Additional File 15: Classifications of *Vitis* proposed by six major systematists between 1895 and 1991

Modified from Galet [1]

	Series	Name used by Moore [2, 3]. Name used by original author in parentheses
**Planchon (1895) in [1]**		
1st Section :	*Muscadinia*	*V. rotundifolia var rotundifolia (V. rontundifolia), V. rotundifolia var munsonia (Munsoniana)*
2nd Section:	*Euvitis*	
Series 1:	*Labruscae*	*V, labrusca*
Series 2:	*Labruscoidaeae american*	*V. californica, V. tilifolia (caribaea), V. shuttleworthii (coriacea), V. mustangensis (candicans).*
Series 3:	*Aestivales*	*V. aestivalis var. linsecumii (Linsecomii), V. aestivales var. bicolor (bicolor), V. aestivalis.*
Series 4:	*Cinerascentes*	*V. cinerea, V . cinerea var helleri (Berlandieri), V. acerifolia (cordifolia).*
Series 5:	*Rupestres*	*V. monticola, V. rupestris, V. arizonica.*
Series 6:	*Ripariae*	*V. riparia, V. palmata (rubra).*
Series 7:	*Labruscoidaeae asiaticae*	*V. coignetiae, V. romanetii, V. ficifolia (Thunbergi), V. jacquemontii (lanata), V. pedicellata.*
Series 8:	Non-classified	*V. Davidii (Spinovitis Davidi).*
Series 9:	*Viniferae*	*V. vinifera.*
**Munson [4]**		
Section pseudovitis	*Muscadinia*	V. rotundifolia var rotundifolia (V. rontundifolia), V. rotundifolia var munsonia (Munsoniana).
Section Euvitis		
Series 1:	*Labruscae*	*V. labrusca.*
Series 2:	*Coriaceae*	*V. shuttleworthii (coriacea), V. mustangensis (candicans), V. x doaniana, V. x Champinii.*
Series 3:	*Aestivales* (Planchon)	*V. aestivales var. aestivales (lincecumii var glauca, simpsoni), V. aestivalis var. lincecumi (lincecumii), V. aestivales var. bicolor (bicolor).*
Series 4:	*Viniferae*	*V. vinifera, V. Bourquiniana.*
Series 5:	*Cinerascentes*	*V. Blancoii, V. tilifolia (caribaea), V. cinerea, V. cinerea var. Floridana, V. cinerea var. helleri (berlandieri), V. cinerea var. baileyana (Baileyana).*
Series 6:	*Cordifoliae*	*V. vulpina (cordifolia), V. palmata (rubra), V. monticola.*
Series 7:	*Occidentales*	*V. californica, V. girdiana, V. arizonica, V. Treleasei.*
Series 8:	*Precoces*	*V. riparia (vulpina), V. acerifolia (Longii), V. rupestris.*
**Bailey [5]**		
Section or subgenus	*Muscadinia*	*V. rotundifolia var rotundifolia (V. rontundifolia), V. rotundifolia var munsonia (Munsoniana)*
Section or subgenus	*Euvitis*	
Series 1:	*Labruscoideae*	*V. labrusca (labrusca and labruscana), V. mustangensis (candicans), V. Shuttleworthii*
Series 2:	*Aestivales*	*V. aestivalis var. aestivalis (Smalliana, Simpsoni, rufotomentosa), V. aestivalis var lincecumii (Lincecumii), V. aestivalis var bicolor (argentifolia), V. aestivalis, V. Bourquina, V. tilifolia (sola).*
Series 3:	*Arachnoideae*	*V. californica, V. girdiana, V. arizonica glabra, V. cinerea var. cinerea (var. canescens) , V. X champinii (V. Champini), V. X doaniana (V. doaniana).*
Series 4:	*Cordifoliae*	*V. vulpina (ilex, cordifolia, cordifolia var. foetida), V. palmata, V. cinerea var. baileyana (Baileyana), V. cinerea var. helleri (berlandieri, Helleri).*
Series 5:	*Vulpinae*	*V. rupestris, V. acerifolia (Longii, longii var. microsperma), V. monticola, V. Treleasei, V. riparia (vulpina), V. X novae-angliae (V. novae-anglia), V. riparia X bicolor (Slavinii), V. Andersonii.*

	Series	Name used by Moore [2, 3]. Name used by original author in parentheses
**Galet [1]**		
Section	*Muscadinia*	*V. rotundifolia var. rotundifolia (V. rotundifolia), V. rotundifolia var. Munsoniana (V. munsoniana), V. Popenoi.*
Section	*Vitis*	
Series 1:	*Candicansae*	*V. mustangensis (candicans), V. doaniana, V. acerifolia (Longii), V. Shuttleworthii (coriacea), V. aeativalis var aestivalis (Simpsonii), V. X champinii (V. Champinii).*
Series 2:	*Labruscae*	*V. labrusca, V. Coignetiae.*
Series 3:	*Caribaeae*	*V. tilifolia (caribaea), V. Blancoii, V. jaquemontii (lanata).*
Series 4:	*Arizonae*	*V. arizonica, V. californica, V. girdiana, V. Treleasei.*
Series 5:	*Cinereae*	*V. cinerea, V. cinerea var. helleri (Berlandieri), V. cinerea var. baileyana (Baileyana), V. Bourgeana.*
Series 6:	*Aestivalae*	*V. aestivalis, V. aetivalis var. bicolor (bicolor), V. aestivalis var. lincecumi (Lincecumii), V. Bourquina, V. aestivalis var. aestivalis (gigas, rufotomentosa).*
Series 7:	*Cordifoliae*	*V. vulpina (cordifolia, ilex), V. cinerea var. helleri (Helleri), V. monticola, V. palmata (rubra)*
Series 8:	*Flexuosae*	*V. flexuosa, V. ficifolia (Thunbergii), V. betulifolia, V. reticulata, V. amurensis, V. Piasezkii, V. Embergeri, V. pentagona, V. chunganensis, V. Chungii, V. piloso-nerva, V. balansaeana, V. Hancockii, V. hexamera, V. pedicellata, V. retordii, V. Seguinii, V. Silvestrii, V. Tsoii, V. bryonifolia, V. pseudoreticulata, V. ficifolioides, V. Retordii, V. hexamera, V. Seguinii, V. wenchowensis, V. chrysobotrys.*
Series 9:	*Spinosae*	*V. armata, V. Davidii, V. Romanetii.*
Series 10:	*Ripariae*	*V. riparia, V. rupestris.*
Series 11:	*Viniferae*	*V. vinifera, V. silvestris.*
**Moore [2,3]**		
Subgenus *Muscadinia*		*V. rotundifolia var. rotundifolia, V. rotundifolia var. munsonia.*
Subgenus *Vitis*		
Series 1:	*Aestivales* (Planchon)	*V. aestivales var aestivales, V. aestivales var. bicolor, V. aestivales var. lincecumii*
Series 2:	*Cinerescentes* (Planchon)	*V. cinerea var. cinerea, V. cinerea var. floridana, V. vinerea var. baileyana, V. cinearea var. helleri*
Series 3:	*Cordifoliae* (Munson)	*V. vulpina, V. palmata, V. monticola*
Series 4:	*Labruscae* (Planchon)	*V. labrusca, V. shuttleworthii, V. mustangensis*
Series 5:	*Ripariae* (Munson)	*V. acerifolia, V. riparia, V. rupestris*
	Hybrids	*V. X Champinii, V. x doaniana, V x novae-angliae*
**Comeaux [6]**	*Aestivales*	*V. nesbittiana*
**Comeaux [7]**	*Occidentales*	*V. bloodworthiana*

1. Galet P: *Cepages et vignobles de France*. *Tome 1*. *Les vignes Americaines.* Dehan: Montpellier; 1988.
2. Moore MO: **Classification and systematics of Eastern North American *Vitis* L. (Vitaceae) north of Mexico**. *SIDA* 1991, **14**:339-367.
3. Moore M.O. **Vitaceae**. In: *Flora of North America North of Mexico*. Edited by Flora of North America Editorial Committee. *Flora of North America:* Cambridge; [unpubl.]
4. Munson TV: *Foundation of American grape culture*. TV Munson and Son: Denison, TX; 1909.
5. Bailey LH: **The species of grapes peculiar to North America**. *Gentes Herbarum* 1934, **3**:154-243.
6. Comeaux BL: **A new *Vitis* (Vitaceae) from Vera Cruz, Mexico**. *SIDA* 1987, **12**:273-277.
7. Comeaux BL: **Two new *Vitis* (Vitaceae) from mountainous Mexico**. *SIDA* 1991, **14**:459-466.
