# Supplementary material for: A phylogenetic analysis of the grape genus (Vitis L.) reveals broad reticulation and concurrent diversification during neogene and quaternary climate change
Source: BMC Evol Biol. 2013 Jul 5;13:141. doi: 10.1186/1471-2148-13-141 (PMC3750556; doi:10.1186/1471-2148-13-141)
Supplement: Additional file 17 — Laboratory procedures.pdf. PCR and cycle sequencing protocols. [file 1471-2148-13-141-S17.pdf]

Laboratory procedures.

PCR reactions for primer screening were performed in 25 µL vol with 20 ng DNA, 3.6-4.5 mM $MgCl_2$, 0.2 mM each dNTP, 8 pmol each forward and reverse primer, 0.25 U GoTaq DNA polymerase, 1x commercial GoTaq reaction buffer (Promega, Madison, WI). PCR volume for exploratory sequencing was scaled up to 50 µL but using the same 20 ng DNA per reaction and 12 pmol of each primer. The PCR conditions were: 4 min 94°C initial denaturation, followed by 35 cycles of 94°C for 1 min, 42-56.8°C for 1 min, 72°C for 2 minutes, with a final extension of 7 min at 72°C and storage at 4°C.

The 50 µL PCR products were cleaned using the Edge Biosystems QuickStep™ 2 PCR purification Kit. A few PCR products were gel purified using the QIAquick gel extraction kit (Qiagen) and concentrated to 12 µL for cycle sequencing.

Cycle sequencing reactions were 12 µL volume with 5.0 µL concentrated PCR products, 3.6 µL 2.5x sequencing buffer (400 mM Tris, pH9 and 10 mM $MgCl_2$; filter sterilized), 6.6 pmol forward or reverse primer, and 1.0µL BigDye Ready Reaction mix version 3.0 or 3.1 (Applied Biosystems). The cycling conditions for sequencing were: 95°C for 3 min, followed by 50 cycles of 96°C for 10 sec, 58°C for 4 min, plus 72°C for 7 min and stored at 4°C. Three uL water were added to each reaction, then products from the cycle sequencing reaction were cleaned using the Performa® DTR 96 well short or standard plates (depending on the version of BigDye; EdgeBioSystems), vacuum dried, and resuspended in 10 µl HiDi™ formamide (Applied Biosystems).

www.edgeBio.com

www.Qiagen.com

www.appliedbiosystems.com
