# Supplementary material for: A phylogenetic analysis of the grape genus (Vitis L.) reveals broad reticulation and concurrent diversification during neogene and quaternary climate change
Source: BMC Evol Biol. 2013 Jul 5;13:141. doi: 10.1186/1471-2148-13-141 (PMC3750556; doi:10.1186/1471-2148-13-141)
Supplement: Additional file 21 — Gene partitions for the 11437bp matrix. [file 1471-2148-13-141-S21.pdf]

**Additional File 21.**

**Gene partitions for the 11437bp matrix**.

The second range of nucleotides are characters added by indel coding.

11736= area code for ancestral area optimization (in 273 OTU matrix)

Starting at 1 (TNT begins counting at 0)

DNA, gene1165 = 1-539, 11438-11450
DNA, gene1313 = 540-1050, 11451-11471
DNA, gene1314 = 1051-1702, 11472-11495
DNA, gene1529 = 1703-2039, 11496-11502
DNA, gene1973 = 2040-2575, 11503-11507
DNA, gene2129 = 2576-3056, 11508-11509
DNA, gene2415 = 3057-3706, 11510
DNA, gene241 = 3707-4126, 11511-11546
DNA, gene3221 = 4127-4491, 11547-11570
DNA, gene3389 = 4492-4867, 11571-11576
DNA, gene5069 = 4868-5437, 11577-11585
DNA, gene5693 = 5438-5803
DNA, gene590 = 5804-6097, 11586-11588
DNA, gene6054 = 6098-6374
DNA, gene6670 = 6375-6776, 11589
DNA, gene689 = 6777-7081, 11590
DNA, gene7022 = 7082-7385, 11591
DNA, gene7029 = 7386-7876, 11592-11618
DNA, gene7230 = 7877-8262, 11619-11621
DNA, gene7312 = 8263-8576, 11622
DNA, gene7351 = 8577-8827, 11623-11631
DNA, gene7362 = 8828-9570, 11632-11659
DNA, gene7386 = 9571-9861, 11660
DNA, gene7413 = 9862-10317, 11661-11671
DNA, gene7434 = 10318-10641, 11672-11694
DNA, gene7447 = 10642-10979, 11695-11715
DNA, gene765 = 10980-11437, 11716-11735
