# Supplementary material for: A phylogenetic analysis of the grape genus (Vitis L.) reveals broad reticulation and concurrent diversification during neogene and quaternary climate change
Source: BMC Evol Biol. 2013 Jul 5;13:141. doi: 10.1186/1471-2148-13-141 (PMC3750556; doi:10.1186/1471-2148-13-141)
Supplement: Additional file 24 — Evaluation criteria for BEAST runs.pdf. Evaluation criteria for Tracer files. [file 1471-2148-13-141-S24.pdf]

Additional File 24. Quality Evaluation of BEAST Runs

BEAST runs were analyzed individually and in combination, compatibility was assessed by evaluating the combined traces (with individually determined burnin) for the likelihood and the posterior, comparing means, and by tracking the effective sample size (ESS) statistic. ESS represents "the number of effectively independent draws from the posterior distribution that the Markov chain is equivalent to" (BEAST website http://beast.bio.ed.ac.uk/Increasing_ESSs) and should be well above 200 to represent the posterior distribution well. Traces should have the form of a "hairy caterpillar" without large-scale fluctuations or trends (http://beast.bio.ed.ac.uk/Analysing_BEAST_output).

Drummond AJ, Ho SYW, Rawlence N, Rambault A 2007: A rough guide to BEAST 14. Available at http://beastbioedacuk/Main_Page#A_Rough_Guide_to_BEAST_14
